# Structural Diversity of Peptoids: Tube-Like Structures of Macrocycles

**DOI:** 10.3390/molecules26010150

**Published:** 2020-12-31

**Authors:** Claudine Nicole Herlan, Katharina Sommer, Patrick Weis, Martin Nieger, Stefan Bräse

**Affiliations:** 1 Institute of Organic Chemistry, Karlsruhe Institute of Technology, Fritz-Haber-Weg 6, 76131 Karlsruhe, Germany; claudine.herlan@kit.edu (C.N.H.); katharina_sommer@posteo.de (K.S.); 2 Institute of Physical Chemistry, Karlsruhe Institute of Technology, Kaiserstr. 12, 76131 Karlsruhe, Germany; patrick.weis@kit.edu; 3 Department of Chemistry, University of Helsinki, P.O. Box 55 (A.I. Virtasen aukio 1), FIN-00014 Helsinki, Finland; martin.nieger@helsinki.fi; 4 Institute of Biological and Chemical Systems—Functional Molecular Systems, Karlsruhe Institute of Technology, Hermann-von-Helmholtz-Platz 1, 76344 Eggenstein-Leopoldshafen, Germany

**Keywords:** peptidomimetics, tricyclic peptoids, CuAAC, foldamers

## Abstract

Peptoids, or poly-*N*-substituted glycines, are characterised by broad structural diversity. Compared to peptides, they are less restricted in rotation and lack backbone-derived H bonding. Nevertheless, certain side chains force the peptoid backbone into distinct conformations. Designable secondary structures like helices or nanosheets arise from this knowledge. Herein, we report the copper-catalysed alkyne-azide cycloaddition (CuAAC) of macrocycles to form innovative tube-like tricyclic peptoids, giving access to host–guest chemistry or storage applications. Different linker systems make the single tubes tuneable in size and enable modifications within the gap. An azobenzene linker, which is reversibly switchable in conformation, was successfully incorporated and allowed for light-triggered changes of the entire tricyclic structure.

## 1. Introduction

Fundamental knowledge of the relationship between structure and function is a principal topic in both biological and material science. Rational design of conformationally stable macromolecules gives access to various applications regarding catalysis, host–guest chemistry, and cargo transport. Peptoids, or poly-*N*-substituted glycines, have been shown to form stable secondary structures, such as macrocycles [1,2,3], helices [4,5,6], or even nanosheets [7,8,9,10]. As bioinspired oligomers, peptoids possess antimicrobial characteristics [11,12,13,14,15,16,17,18,19,20,21,22,23] and hold promise for effective pharmaceuticals [20,24,25,26,27]. Attached to catalysts, they can induce enantioselectivity by structure [28,29]. In the form of nanosheets, peptoids act as molecular sensors or artificial membranes [7,8,30,31,32,33,34].

The submonomer method, firstly described by Zuckermann et al. [35], gives access to an infinite number of structurally diverse oligopeptoids, as various amines are easily introduced as sidechains. This remarkable diversity provides the basis for rationally designed functional foldamers that render peptoids a promising substance class for various applications.

**Scheme 1:**
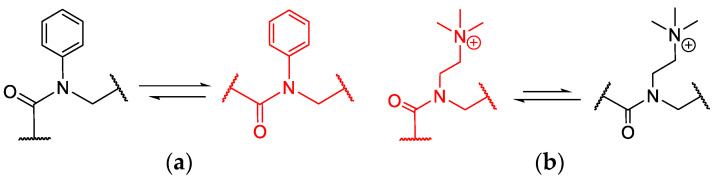
Conformational equilibrium of a peptoid backbone. (**a**) *N*-aryl sidechain enforcing *trans*-conformation [36] and (**b**) positively charged *N*-alkyl sidechain favouring *cis*-amide conformation [40].

Although the peptoid backbone is highly flexible due to its rather free rotating methylene unit, it can be forced into distinct conformations by a selection of certain sidechains. Thereby, aniline derivatives favour *trans*-conformations, while some aliphatic amines *cis*-conformations (Scheme 1) [36,37,38,39,40].

Secondary structures can thus be induced via sequence control [41,42], but the scope of conformational permutations and the influence of certain side chains still have not been fully investigated. To constrain secondary structures, peptoid macrocycles of different sizes have been synthesised [1,2,37,38,43,44,45,46,47,48]. Among them, octameric macrocycles whose spatial structures were determined via X-ray crystallography and nuclear magnetic resonance (NMR) techniques [3,37,38,43,44,48,49]. Cyclic octamers were capable of performing host–guest interactions within their cavity [38,44,49] and could be further modified using copper-catalysed alkyne-azide [3 + 2] cycloaddition (CuAAC) [37,50,51]. CuAAC is a high-yielding, bio-orthogonal method that enables fast and facile modifications [49,50]. Kirshenbaum et al. reported the use of this kind of “click” chemistry to form cyclic peptoids [2,51]. By CuAAC of distinct side chains, the group was, furthermore, able to build up bi- and tricyclic structures [37].

**Figure 1:**
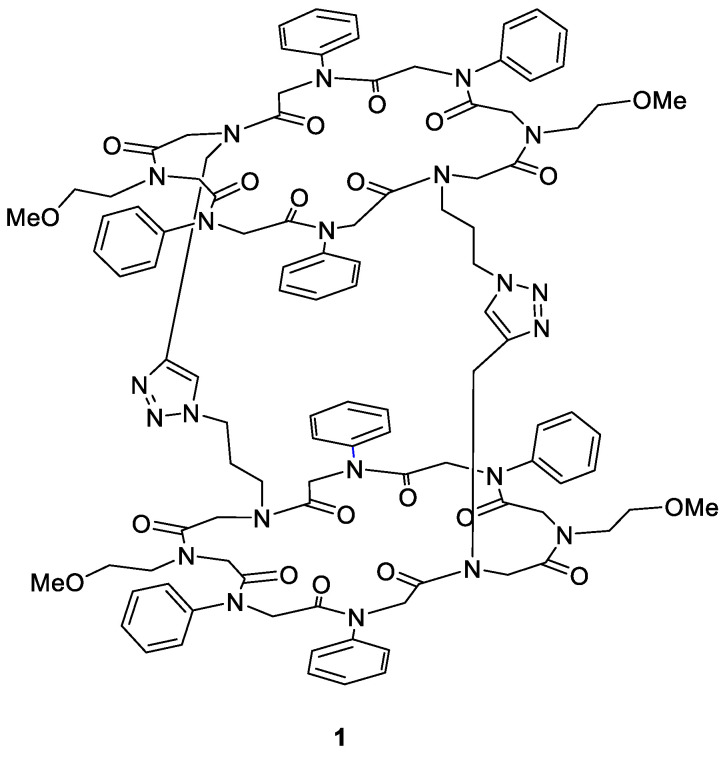
Tricyclic peptoid reported by the group of Kirshenbaum in 2012 [37].

To date, only one of these tube-like tricyclic structures is known (Figure 1). It was built up by CuAAC of two cyclic octamers with one alkyne and one azide moiety each [37]. Herein, we report the design of further rigidified tubular structures with modified linkage structures to enlarge the applicability of these unique peptoid macrocycles.

## 2. Results and Discussion

### 2.1. Design and Synthesis of Cyclic Peptoids

**Scheme 2:**
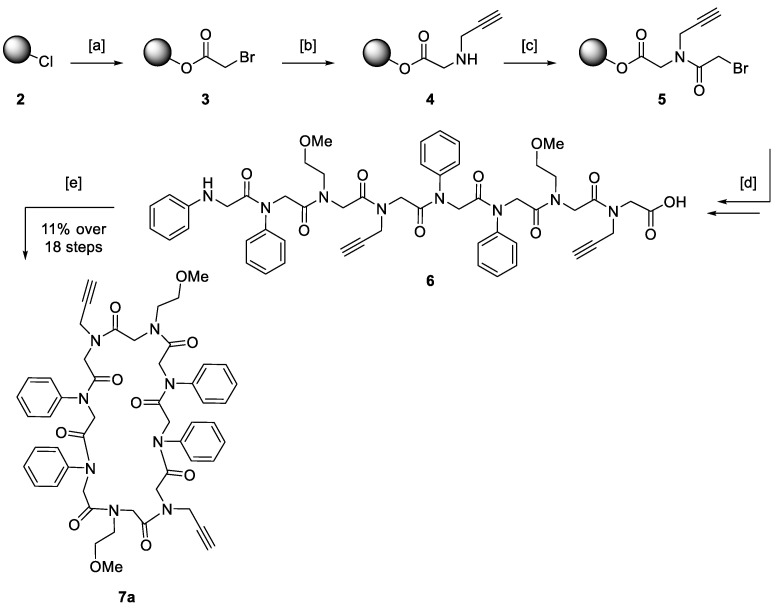
A general approach for the stepwise synthesis of cyclic peptoids as derivatives of model structure **7a**, including solid-phase synthesis on a chlorotrityl chloride resin (**2**) and cyclisation of the linear precursor in solution. (a) Bromoacetic acid, *N*,*N’*-diisopropylethylamine, methylene chloride (DCM), 21 °C, 1 h; (b) propargylamine, *N*,*N’*-dimethylformamide (DMF), 21 °C, 1 h; (c) bromoacetic acid, *N*,*N’*-diisopropylcarbodiimide, DMF, 21 °C, 30 min; (d) (1). Alternating coupling of a desired amine following (b) and bromoacetic acid following (c); (2). hexafluoroisopropanol, DCM, 21 °C, 30 min; (e) PyBOP, DIPEA, dry DCM, 21 °C, 16 h.

The stepwise synthesis of peptoid macrocycles was carried out using the published submonomer method on solid support [35]. Acetylation and substitution steps were alternated until the desired chain length was achieved. After cleavage from the resin, linear precursors were converted in the solution without further purification. Cyclisation was carried out according to a protocol by Kirshenbaum et al. [1] without exceeding the peptoid concentration of 4.80 mM to circumvent dimerisation (Scheme 2).

By combining both solid- and liquid-phase techniques, plenty of peptoid macrocycles were readily accessible. Alkyne and azide moieties were incorporated to give access to post-synthetic modifications via CuAAC [50].

Peptoids were designed with the objective of the final backbone topology *c-c-t-t-c-c-t-t*, where “*c*” indicates *cis*-conformation and “*t*” indicates *trans*-conformation of the single-peptoid subunits. In our previous study, structural data of a macrocyclic octamer similar to peptoid **7a** that showed the desired backbone conformation were obtained [38]. Thereby, incorporation of *N*-aryl amines stabilised the *trans*-amide structure elements. Different *N*-alkyl amines gave access to *cis*-conformations, ensuring the spatial arrangement required for cyclisation. Based on these former results, peptoid **7a** was contrived to represent the model structure of every macrocycle synthesised.

Considering further cycloaddition reactions, each peptoid was functionalised with either two alkyne or azide moieties. This design should enable the combination of two different macrocycles to form a tricyclic structure via intermolecular CuAAC of their sidechains. Functional amines capable of the click reaction were both aliphatic and aromatic. Four different cyclic peptoids (**7a**–**7d**, Table 1) were synthesised according to the general approach described in Scheme 2. The cyclic octamers consist of four aryl and alkyl building blocks each. The respective monomers were arranged pairwise to ensure the desired backbone topology. Macrocycles **7a** and **7b** contain two adjacent anilines, as well as aliphatic functionalities as capable moieties for cycloaddition next to a methoxyethyl residue. Peptoids **7c** and **7d** are decorated with *para*-substituted anilines and two adjacent methoxyethyl sidechains.

Purification was carried out via preparative reverse-phase high performance liquid chromatography (HPLC) solely after the cyclisation step. Product formation was confirmed via matrix-assisted laser desorption/ionization-time of flight (MALDI-TOF) measurements. Macrocycles **7a**–**7d** were obtained in moderate yields (8.0% to 25%) over 18 steps each. Purity was determined by integration of analytical reverse-phase HPLC traces and turned out to account for 87% to 99%.

### 2.2. Topology of Cyclic Peptoids

A precondition for successful CuAAC of two macrocycles is a defined spatial arrangement of the alkyne and azide moieties. Both structural elements have to point upward or downward on the backbone, but necessarily in the same direction. Only one 3D structure of macrocyclic octamers resembling the synthesised ones is known from the literature [38]. Vollrath et al. published crystallographic data of a peptoid similar to macrocycle **7a** in 2013. The building blocks of the published octamer comply with the ones of structure **7a**, but the positions of both the two aliphatic alkyne side chains and the methoxyethyl residues are interchanged. The backbone of this similar macrocycle showed the desired topology *c-c-t-t-c-c-t-t*, with sidechains pointing alternatingly up and down and both alkyne moieties located on the same side of the ring level.

**Figure 2:**
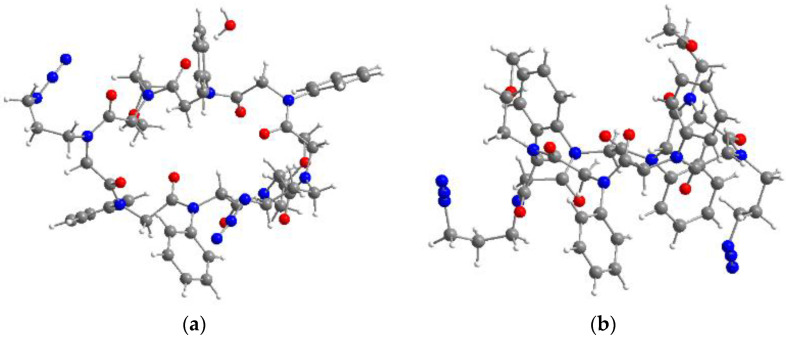
Molecular structure of cyclic peptoid **7b** revealing the backbone topology *c-c-t-t-c-c-t-t* with both aliphatic azides located on the same side of the ring level. (**a**) Top view; (**b**) side view.

It was assumed that peptoid **7a** would be arranged in the same topology due to structural analogy. Unfortunately, no measurable crystals of **7a** were obtained. However, macrocycle **7b**, equipped with two aliphatic azides instead of the alkyne moieties, was successfully crystallised. X-ray diffraction experiments revealed its stabilised structure (Figure 2).

Comparison of the structural data of the previously published octamer and peptoid **7b** disclosed an equal backbone topology, including identical angles [38]. The sidechains are alternatingly located above and below the ring level, with both azide moieties pointing in the same direction. In contrast to propargylamine, the functional group of azidopropylamine is separated by three methylene units. The enlargement of conformational freedom by rotation around carbon single bonds makes the latter more flexible.

**Figure 3:**
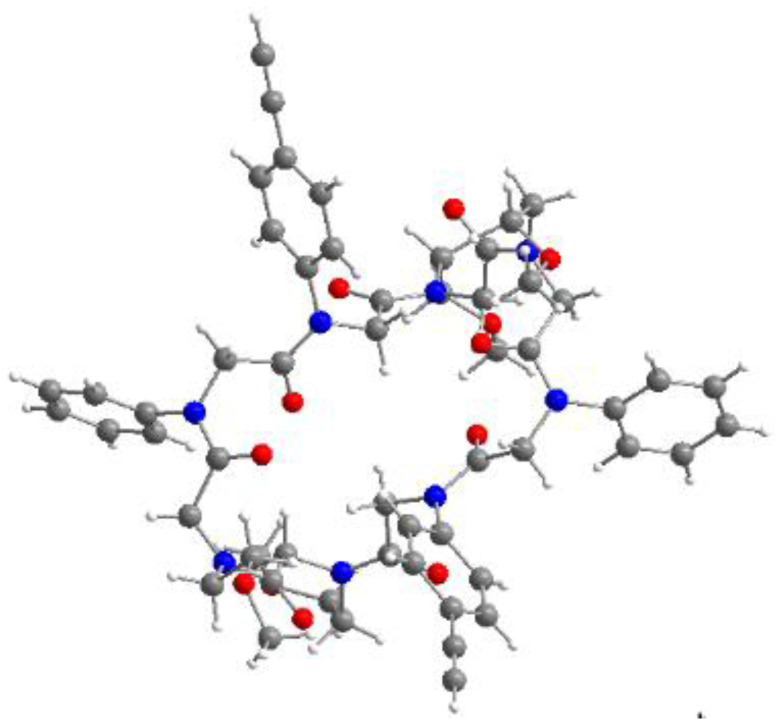
Molecular structure of one of the two independent molecules of the cyclic peptoid **7c** decorated with two aromatic alkynes. The structural data coincide with those obtained for macrocycle **7b**.

For cyclic octamers with aromatic alkyne or azide moieties, like peptoids **7c** and **7d**, neither conformational data nor information about the orientation of both functionalisable groups have been found so far. Measurable crystals of peptoid **7c** were obtained after multiple attempts, and their structure was elucidated via X-ray diffraction (Figure 3).

The backbone topology equals the one known from other eight-membered macrocycles [1,38]. Again, the side chains are alternatingly located above and below the ring level, resulting in both alkyne moieties pointing into the same direction. These data coincide with those of the cyclic octamers **7a** and **7b** [38]. Due to the aromatic systems introduced, the functionalisable groups were rigidified compared to derivatives decorated with aliphatic moieties.

Although no measurable crystals of macrocycle **7d** could be obtained, a similar conformation was expected due to the strong structural similarities to the remaining three macrocycles **7a**–**7c**.

Every crystallised compound revealed the desired backbone topology *c-c-t-t-c-c-t-t*, as proven for model structure **7a** [38]. Aliphatic and aromatic azides, as well as alkynes, were successfully incorporated, and the design allowed for a location on the same side of the ring level. The latter constitutes a prerequisite for further modifications via CuAAC to design tricyclic tube-like structures.

### 2.3. CuAAC of Two Cyclic Peptoids

Copper-catalysed alkyne-azide cycloaddition is an effective method for crosslinking different structures. The four macrocycles mentioned were conjugated pairwise via CuAAC following a modified protocol by Jagasia et al. [52]. Catalysis was performed with tetrakis(acetonitrile)copper(I) hexafluorophosphate (Cu(CH_3_CN)_4_PF_6_) and 2,6-lutidine. The reaction was carried out in dry methylene chloride under inert conditions. Monitoring of the product formation via analytical reverse-phase HPLC revealed a reaction time of three days for every starting material to be converted. Subsequent purification was carried out via preparative reverse-phase HPLC.

**Scheme 3:**
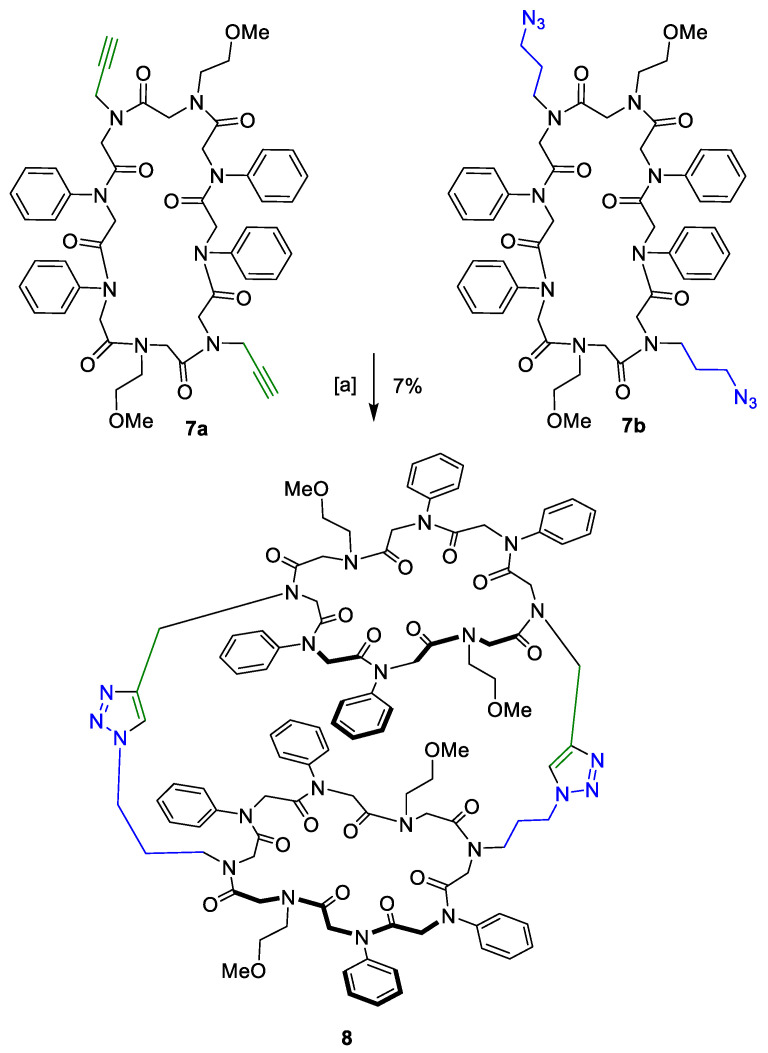
CuAAC of peptoids **7a** and **7b** decorated with aliphatic functional groups. (a) 2,6-Lutidine, Cu(CH_3_CN)_4_PF_6_, dry DCM, 21 °C, 3 days.

CuAAC of peptoids **7a** and **7b**, decorated with aliphatic functional groups, yielded the tricyclic compound **8** (Scheme 3).

The tube-like structure was isolated in 7% yield. Analysis via MALDI-TOF and analytical HPLC revealed product formation with a purity of more than 99%. To validate conversion, IR measurements of both the azide-functionalised starting material and the final tricycle were taken. The characteristic azide band visible in the IR trace of peptoid **7b** was omitted in the product spectrum. This measurement verified the formation of the tricyclic structure with both azide moieties converted into a triazole.

**Scheme 4:**
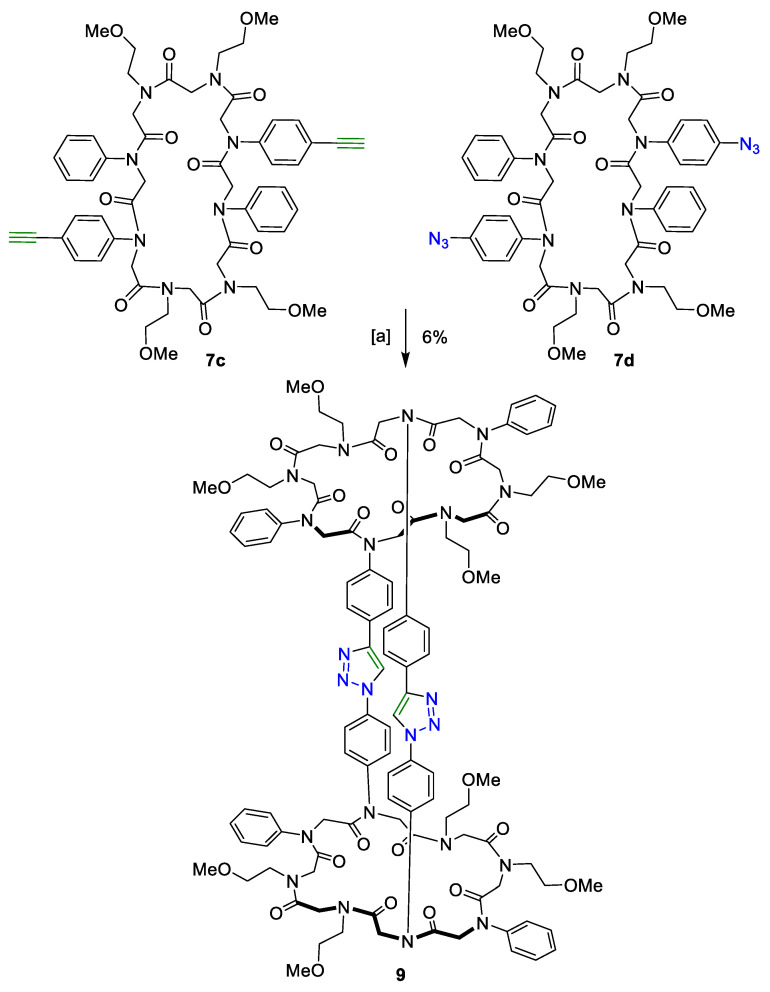
Tricycle **9** as a product of the CuAAC of peptoids **7c** and **7d** decorated with aromatic functional groups. (a) 2,6-Lutidine, Cu(CH_3_CN)_4_PF_6_, dry DCM, 21 °C, 3 days.

The CuAAC of the two macrocycles decorated with aromatic alkynes and azides (**7c** and **7d**) was carried out following the procedure mentioned above. Product **8** was isolated in 6% yield and 98% purity (Scheme 4).

The yields for the CuAAC of two aliphatic and of two aromatic residues are similar. Therefore, it is reasonable that neither flexibility nor rigidity is advantageous for this kind of reaction.

### 2.4. CuAAC of Cyclic Peptoids and Small Molecules

**Scheme 5:**
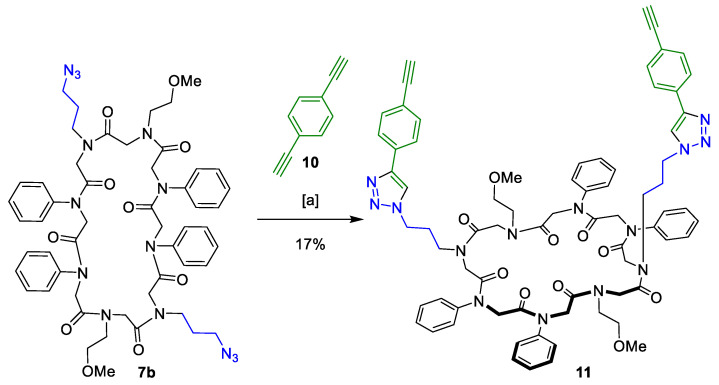
CuAAC of the macrocyclic peptoid **7b** and 1,4-diethynylbenzene (**10**). (a) 2,6-Lutidine, Cu(CH_3_CN)_4_PF_6_, dry DCM, 21 °C, 3 days.

To increase the structural diversity of the tube-like tricyclic peptoids, small molecules were incorporated by tuning the size of the tube and allowing access to further functionalisation. For size enlargement of the cavity in between the macrocycles, 1,4-diethynylbenzene (**10**) was attached to peptoid **7b** (Scheme 5).

The bifunctionalised linker **10** was added in excess to enable full conversion and to circumvent intramolecular crosslinking. The reaction conditions complied with the ones mentioned for the CuAAC of the two peptoid macrocycles. Compound **11** was isolated in 17% yield after purification. Product formation was verified via MALDI-TOF and purity was determined to be 99%. The CuAAC of linker conjugate **11** and another azide-functionalised peptoid macrocycle failed.

To enable further structural diversity that was adjustable via light, an azobenzene linker system was synthesised according to a published procedure [53]. Azobenzenes are known for their ability to switch their conformation from *cis* to *trans* and vice versa after irradiation with light [54,55]. They have been used as submonomers in solid-phase synthesis and have been shown to affect the structure of the resulting peptoid derivatives due to triggerable *cis-trans*-isomerisation [56].

**Scheme 6:**
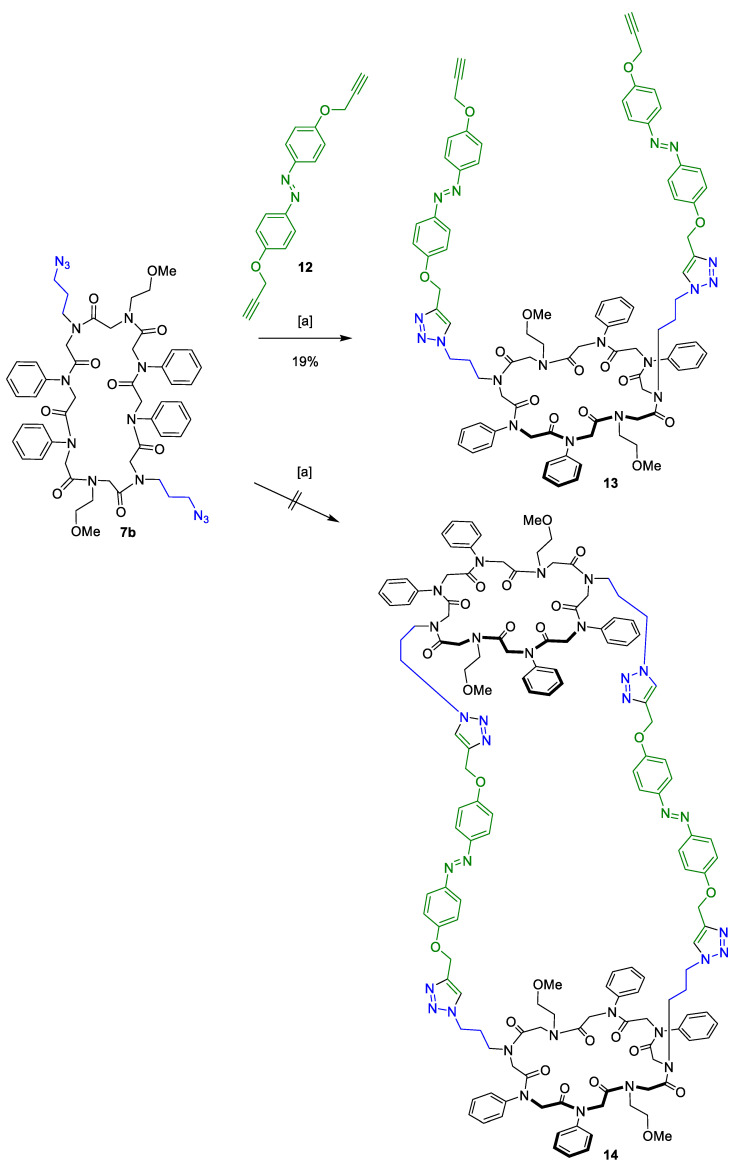
CuAAC of the cyclic peptoid **7b** and the azobenzene linker **12** revealed conjugate **13**, but did not form the tricyclic structure **14**. (a) 2,6-Lutidine, Cu(CH_3_CN)_4_PF_6_, dry DCM, 21 °C, 3 days.

The effectivity of the conformational change after UV radiation was monitored via NMR for the synthesised linker (see Supplementary Materials). The potent azobenzene should then be incorporated as a spacer between two macrocycles. So far, examples for only linear peptoids with an azobenzene switch have been found [56]. Herein, we report the first peptoid macrocycle attached to an azobenzene. Conjugate **13** (Scheme 6) was synthesised via CuAAC according to the conditions mentioned above. Product **13** was obtained in 19% yield and 98% purity, similarly to the yield obtained for the CuAAC of the simplified linker **10** (Scheme 5).

**Figure 4:**
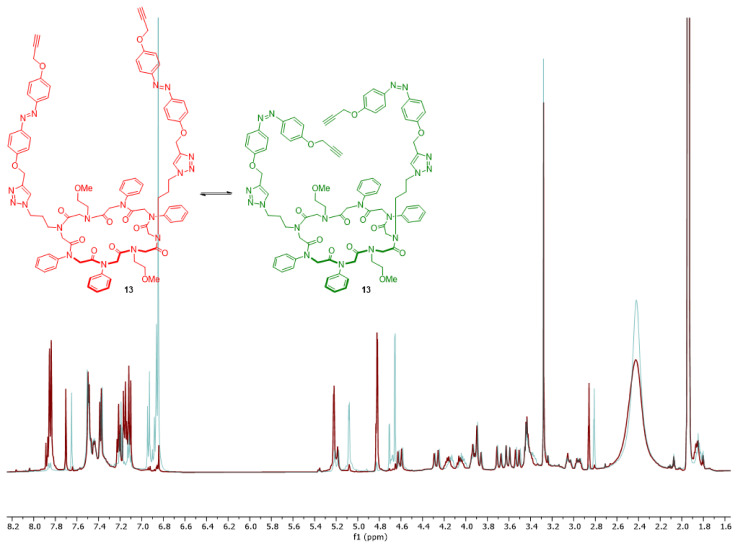
Switch experiment of conjugate **13**, whose conformation change from *trans* (red) to *cis* (green) after irradiation with UV light was monitored via nuclear magnetic resonance (NMR).

The subsequent switch experiments should confirm the conformational change of the entire system **13**. NMR spectra of product **13** both before and after irradiation with UV light were measured (Figure 4).

Significant changes in the aromatic area were observed. Additionally, the signals of the terminal alkyne and methylene groups of the azobenzene linkers shifted. UV-Vis measurements verified these results (see Supplementary Materials). Therefore, the azobenzene of conjugate **13** can switch efficaciously from *trans*- to *cis*-conformation after irradiation with UV light.

Unfortunately, the desired tricyclic structure **14** with one peptoid macrocycle attached to each alkyne moiety of the azobenzene linker could not be isolated (Scheme 6). It is conceivable that the chosen linker system is unable to close the tricyclic tube via CuAAC due to its high degree of flexibility. Compound **13** polymerised to an insoluble solid instead.

To circumvent polymerisation, a rigidified azobenzene linker system was synthesised according to a procedure known from the literature [57]. Switch experiments revealed conformational changes with effectivities of 97% for *trans*- and 95% *cis*-conformers (see Supplementary Materials).

**Figure 5:**
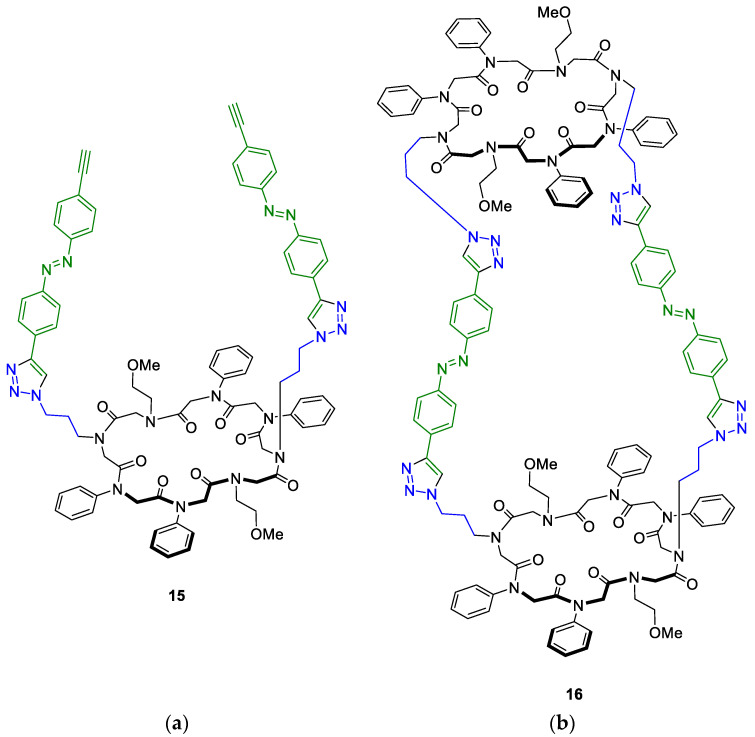
Products of the CuAAC of peptoid **7b** and a rigidified azobenzene linker (green). (**a**) Conjugate **15** displays the linker with one peptoid macrocycle attached to one alkyne moiety each. (**b**) CuAAC of both alkyne moieties led to the tube-like structure **16**.

The CuAAC of the rigidified linker and peptoid **7b** was carried out as described before, using an excess of the azobenzene linker to circumvent intramolecular crosslinking. After three days, macrocycle **15** and the tube-like structure **16** were isolated (Figure 5).

Structure **15** with peptoid conjugation to one alkyne moiety of the azobenzene linker was obtained in 3% yield after purification. Interestingly, the yield is significantly less compared to the CuAAC of the more flexible azobenzene linker **12** described above. In return, the desired tube-like tricyclic peptoid structure **16** was additionally obtained in a similar yield of 2%. Masses of both products were confirmed via MALDI-TOF and electrospray ionization (ESI-TOF). NMR measurements verified the receipt of the single structures (see Supplementary Materials).

**Figure 6:**
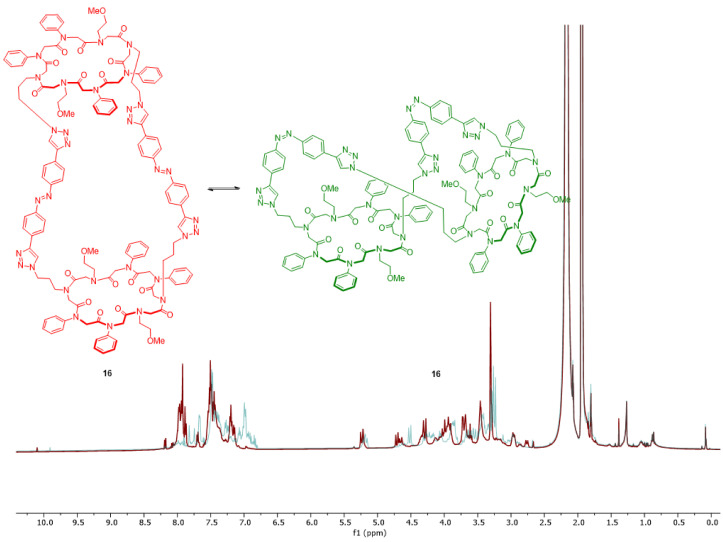
Switch experiment of the tricyclic structure **16,** whose conformation change from *trans* (red) to *cis* (green) after irradiation with UV light was monitored via NMR.

Switch experiments of both molecules were monitored via NMR after irradiation with UV light (365 nm) for 30 min. The conformational change from *trans* to *cis* was verified by significant shifts in the aromatic region (Figure 6). In the case of product **15**, an additional signal shift of the terminal alkyne proton was observable after irradiation (see Supplementary Materials).

**Figure 7:**
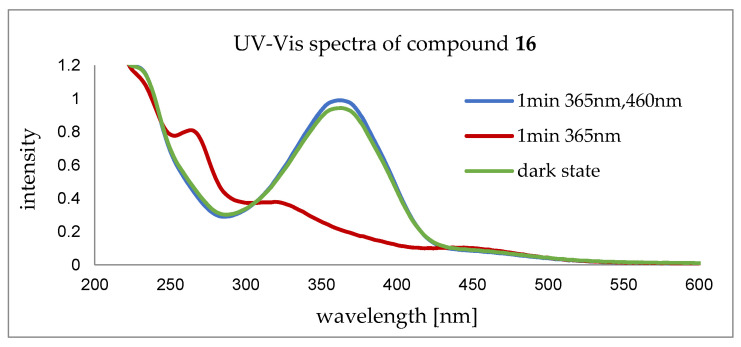
UV-Vis spectra of compound **16** before (green) and after irradiation with UV light (365 nm) for 1 sec (red), indicating a conformational change from *trans* to *cis*. The reverse conformational change was induced via irradiation with UV light (365 nm), followed by irradiation with visible light (460 nm) for 1 min each (blue).

Additionally, UV-Vis spectra of compounds **15** and **16** were recorded before and after irradiation with UV light (Figure 7 and Supplementary Materials).

*Trans*-conformation is indicated by the dark state trace. Conformational change towards the *cis*-conformation was triggered by irradiation with UV light (365 nm) for 1 min. Reverse conformational change from *cis* to *trans* was monitored after irradiation with UV light (365 nm) first to force the azobenzene into *cis*, with subsequent irradiation with visible light (460 nm) for 1 min each.

When changing conformation from *trans* to *cis*, a significant increase in intensity is observable at around 280 nm. A decrease occurs simultaneously at around 360 nm. The trace of the probe irradiated with UV light first and with visible light next corroborates the transfer from *cis* to *trans*, showing the reversibility of the system.

These results give evidence of a reversible conformational switch of the tricyclic peptoid **16**. With this, the tube-like structure can open and close, rendering it applicable for storage or host–guest chemistry.

## 3. Materials and Methods

General procedure for the synthesis of cyclic peptoids: In a fritted syringe, a 2-chlorotrityl-chloride resin (300 mg, 0.480 mmol, 1.60 mmol/mg loading density, 100–200 mesh, 1.00 equiv.) was swollen in 3 mL of methylene chloride (DCM) for 2 h. After filtration, a freshly prepared solution of bromoacetic acid (2.59 mmol, 5.40 equiv.) and *N*,*N*′-diisopropylethylamine (DIPEA, 2.59 mmol, 5.40 equiv.) in 2.5 mL of DCM was added and shaken for 1 h at 21 °C. The resin was extensively washed with peptide-grade *N*,*N*′-dimethyl-formamide (*p*DMF). For the following substitution reaction, a solution of the corresponding amine (3.98 mmol, 8.30 equiv.) in 2.5 mL of *p*DMF was added to the resin and shaken for 30 min at room temperature (overnight in the case of aniline). Following extensive washing with *p*DMF, a solution of bromoacetic acid (4.80 mmol, 10.0 equiv.) and *N*,*N*′-diisopropylcarbodiimide (DIC, 4.80 mmol, 10.0 equiv.) in 2.5 mL *p*DMF were added and shaken for 30 min at room temperature (2 h in the case of aniline). The acetylation and substitution steps were alternated repeatedly until the desired peptoid length was achieved. For cleavage, a solution of 33% hexafluoroisopropanol in DCM was added and the mixture was shaken overnight. The solvent was removed under reduced pressure.

For cyclisation, the activating agent benzotriazol-1-yl-oxytripyrrolidino-phosphonium hexafluorophosphate (PyBOP, 1.44 mmol, 3.00 equiv.) and DIPEA (2.88 mmol, 6.00 equiv.) were added to a solution of the respective peptoid in 100 mL of DCM. The solution was stirred overnight at room temperature, and the solvent was removed under reduced pressure. The residue was dissolved in a mixture of acetonitrile/water (1:1), lyophilised, and purified via preparative reverse-phase HPLC.

General procedure for the CuAAC of the two peptoids: Both the peptoid decorated with azide (1.00 equiv.) and the one decorated with alkyne moieties (1.00 equiv.) were dissolved in dry DCM and degassed with argon. Afterwards, 2,6-lutidine (6.00 equiv.) was added and the solution was stirred for 5 min. Finally, tetrakis(acetonitrile)copper(I) hexafluorophosphate (Cu(CH_3_CN)_4_PF_6_, 1.00 equiv.) was added, and the mixture was stirred for 3 days at room temperature. The solvent was removed under reduced pressure and the residue was purified via preparative reverse-phase HPLC.

General procedure for the CuAAC of a linker and a peptoid: The desired peptoid (1.00 equiv.) and the respective linker (10.0 equiv.) were dissolved in dry DCM and degassed with argon for 10 min. Afterwards, 2,6-lutidine (8.00 equiv.) was added and the solution was stirred for 5 min. Finally, Cu(CH_3_CN)_4_PF_6_ (1.00 equiv.) was added and the mixture was stirred for 3 days at room temperature. The solvent was removed under reduced pressure and the residue was purified via preparative reverse-phase HPLC.

Switch experiments via NMR: The sample was dissolved in deuterated acetonitrile and stored under exclusion of light. Measurements were performed on a Bruker 500 spectrometer at 500 MHz. Spectra were recorded before and after irradiation with UV light (365 nm).

Switch experiments via UV-Vis: 20 μM solutions of the single samples were stored in the dark. Measurements were performed on a PerkinElmer Lambda 750 UV/Vis/NIR spectrometer and monitored in the range from 200 to 800 nm wavelength. Spectra were monitored before and after irradiation with UV and visible light, respectively.

### Crystal Structure Determinations

The single-crystal X-ray diffraction study was carried out on a Bruker D8 Venture diffractometer with a Photon100 detector at 123(2) K (**7b**) and an Agilent SuperNova-Dual diffractometer with an Atlas CCD-detector at 120(2) K (**7b**) using Cu-Kα radiation (*λ* = 1.54178 Å). (Bruker AXS GmbH, Karlsruhe, Germany] Agilent Technologies, Oxford, United Kingdom) Dual-Space Methods (SHELXD) (for **7b**) or Direct methods (SHELXS-97) (for **7c**) (SHELXD and SHELXS [58]) were used for the structure solution, and refinement was carried out using SHELXL-2013 or SHELXL-2014 (full-matrix least-squares on *F*^2^) (SHELXL [59]). Hydrogen atoms were refined using a riding model (H(water) free). Semi-empirical absorption corrections were applied. For **7b**, an extinction correction was applied. In **7b**, the solvent water and the two 3-azidopropyl groups were disordered (see cif-file for details). In **7c**, the absolute structure could not be determined reliably (Parsons x-parameter, x = 0.5(2); see cif-file for details) [60].

**7b**: colourless crystals, C_52_H_62_N_14_O_10_ − 0.5 H_2_O, *M*_r_ = 1052.16, crystal size 0.36 × 0.12 × 0.06 mm, monoclinic, space group *C2/c* (No. 15), *a* = 23.0990(10) Å, *b* = 18.4326(8) Å, *c* = 25.4929(12) Å, *β* = 105.159(2)°, *V* = 10476.5(8) Å^3^, *Z* = 8, *ρ* = 1.334 Mg/m^−3^, *µ*(Cu-K_α_) = 0.789 mm^−1^, *F*(000) = 4456, *2θ*_max_ = 145.4°, 50,224 reflections, of which 10,350 were independent (*R*_int_ = 0.073), 687 parameters, 51 restraints, *R*_1_ = 0.090 (for 8336 I > 2σ(I)), w*R*_2_ = 0.228 (all data), *S* = 1.04, largest diff. peak/hole = 1.196 (in disordered 3-azidopropyl)/−0.772 e Å^−3^.

**7c**: colourless crystals, C_56_H_64_N_8_O_12_, *M*_r_ = 1041.15, crystal size 0.19 × 0.08 × 0.04 mm, orthorhombic, space group *Pca2_1_* (No. 29), *a* = 36.3213(8) Å, *b* = 9.1548(3) Å, *c* = 32.2512(7) Å, *V* = 10724.0(5) Å^3^, *Z* = 8, *ρ* = 1.290 Mg/m^−3^, *µ*(Cu-K_α_) = 0.754 mm^−1^, *F*(000) = 4416, *2θ*_max_ = 152.4°, 24,059 reflections, of which 15,570 were independent (*R*_int_ = 0.043), 1369 parameters, 1 restraint, *R*_1_ = 0.079 (for 12810 I > 2σ(I)), w*R*_2_ = 0.214 (all data), *S* = 1.03, largest diff. peak/hole = 0.851/−0.306 e Å^−3^.

Cambridge Chrystallographic Data Centre (CCDC) 1446764 (**7b**) and 1446765 (**7c**) contain the supplementary crystallographic data for this paper. These data can be obtained free of charge from The Cambridge Crystallographic Data Centre via www.ccdc.cam.ac.uk/data_request/cif.

## 4. Conclusions

Herein, we report the successful synthesis of tube-like tricyclic peptoids via CuAAC, giving access to novel secondary structures. An azobenzene linker system was successfully incorporated as a spacer between two macrocycles. The opportunity to modify the linkage makes the structures tuneable in size and enables the introduction of functional centres. The azobenzene changed its conformation reversibly from *trans* to *cis* after irradiation with UV and visible light, respectively. With this, the tricyclic structure was forced into bot an open and a closed conformation. This switch was verified using NMR experiments and UV-Vis measurements. Hence, this new class of tube-like peptoid structures gives access to host–guest applications or controllable storage of small molecules.

## Figures and Tables

**Table 1:** Synthesised eight-membered cyclic peptoids **7a**–**7d**, capable of copper-catalysed alkyne-azide cycloaddition (CuAAC).

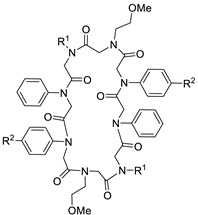				
Macrocycle	R^1^	R^2^	Yield	Purity
**7a**	propargyl	H	11%	99%
**7b**	propylazide	H	25%	98%
**7c**	2-methoxyethyl	ethynyl	8.0%	96%
**7d**	2-methoxyethyl	N_3_	9.0%	87%

## Data Availability

The data presented in this study are openly available in the Chemotion repository: www.chemotion-repository.net.

## Supplementary Materials

The following are available online: synthetic procedure, switch experiments.

## Sample Availability

The molecules are deposited at the Compound Platform of the KIT. Selected molecules are available on request.
