# Supplementary material for: Structural Diversity of Peptoids: Tube-Like Structures of Macrocycles
Source: Molecules. 2020 Dec 31;26(1):150. doi: 10.3390/molecules26010150 (PMC7795174; doi:10.3390/molecules26010150)
Supplement: Supplementary file 1 [file molecules-26-00150-s001.pdf]

Structural Diversity of Peptoids: Tube-like Structures of Macrocycles

Claudine Nicole Herlan, Katharina Peschko, Patrick Weis, Martin Nieger and Stefan Bräse

SUPPORTING INFORMATION

General experimental details

Nuclear Magnetic Resonance Spectroscopy (NMR)

NMR spectra were recorded at 25 °C on a BRUKER Avance 300 (300 MHz (^{1}H), 75 MHz (^{13}C)) and BRUKER 500 (500 MHz (^{1}H), 125 MHz (^{13}C)) spectrometer. Deuterated solvents were purchased from EURISOTOP. Chemical shifts (δ) are expressed in parts per million (ppm) downstream from tetramethylsilane. All spectra are referenced to the signals of the residual protons of the solvents chloroform-d_1 (7.26 ppm (^{1}H), 77.16 ppm (^{13}C)) or acetonitrile-d_3 (1.94 ppm (^{1}H), 1.32 ppm (^{13}C)) as an internal standard. The spectra were analyzed according to first order. Coupling constants (J) are given in Hertz (Hz). Multiplicities of signals are described as follows: s = singlet, bs = broad singlet, d = doublet, t = triplet, q = quartet, quin = quintet, m = multiplet. Abbreviations for signal assignments include H_{Ar} = aromatic proton, C_{Ar} = aromatic carbon, C_q = quaternary carbon.

Mass Spectroscopy (MS)

Electron ionization (EI, 70 eV) mass spectra were measured on a FINNIGAN MAT 90 spectrometer. The molecule fragments are expressed in mass to charge ratio (m/z) and the intensities are given as a percentage relative to the base signal (100%). The molecular ion and protonated molecular ion are referred to as $[M]^+$ and $[M+H]^+$, respectively.
Matrix-assisted laser desorption ionization-time of flight (MALDI-TOF) mass spectra were recorded on a BRUKER Biflex IV spectrometer with a pulsed ultraviolet nitrogen laser (200 µJ at 337 nm) and a time-of-flight mass analyzer with a 125 cm linear flight path. For every spectrum, the samples were shot between 100 and 300 times with a repetition rate of 1–3 Hz. The software WACQ Version 4.0.4 and XMASS_TOF Version 5.1.0 were used for recording and processing of the spectra. The samples were spotted on a BRUKER Standard stainless steel target with 386 spots. As a matrix, a commercially available 1:1 mixture of 2,5-dihydroxybenzoic acid (DHB) and α-cyano-4-hydroxycinnamic acid (CHCA) (Universal MALDI matrix from SIGMA-ALDRICH) as a saturated solution in 50% acetonitrile in water with 0.1% trifluoroacetic acid (TFA) was used. The protonated molecule ion is expressed as $[M+H]^+$.

Infrared Spectroscopy (IR)

IR spectra were measured on a BRUKER IFS 88. Oils were measured as films between KBr plates. Solid samples were measured with the ATR (Attenuated Total Reflection) technique. Wavenumbers (ν) of the absorption bands are given in cm^{-1}. Intensities are

described as follows: vs = very strong (0-10% T), s = strong (11-40% T), m = middle (41-70% T), w = weak (71-80% T) and vw = very weak (91-100% T).

High-performance liquid chromatography (HPLC)

Reverse phase analytical HPLC was performed on an AGILENT Series 1100 equipped with a G1322A-degasser, a G1311A-pump, a G1313A-autosampler, a G1316A oven and a G1315B-diode array detector (DAD). A column, a C18 PerfectSil Target (MZ ANALYSENTECHNIK, 3–5 µm, 4.0 × 250 mm) with a flow rate of 1 mL/min was used. Purity runs were carried out with a gradient of 5–95% acetonitrile in water with 0.1% TFA over 20 min (unless stated otherwise) and the purity was calculated by integration of the signals at 218 nm.

Reverse phase preparative HPLC was performed using a JASCO HPLC of the LC-NetII/ADC Series a Vydac 218TP Series (GRACE DAVISON DISCOVERY SCIENCES), equipped with a C18 column (19 cm × 3 cm), an MD210 Plus multiwavelength detector, PU-2087 Plus pumps, a CO2060 Plus thermostat and a CHF-122SC fraction collector(ADVANTEC). An acetonitrile gradient in water with 0.1% TFA was used as eluent. The flow rates varied between 10–15 mL/min. The method (gradient, temperature and flow rate) was adjusted to each sample.

General experimental procedures

Solvents and reagents were purchased from commercial sources and used without further purification. Abbreviations are as follows: 1,1,1,3,3,3-hexafluoroisopropyl alcohol (HFIP), acetonitrile (ACN), Benzotriazol-1-yl-oxytripyrrolidinophosphonium-hexafluorophosphate (PyBOP), *N,N′*-diisopropylcarbodiimide (DIC), *N,N′*-diisopropylethylamine (DIPEA), peptide grade dimethylformamide (*p*DMF), lithium diisopropylamide (LDA), tetrahydrofuran (THF).

Reagents and products were weighted on SARTORIUS analytical scales, models LA310S and BP211D.

Moisture and air-sensitive reactions were carried out under argon according to the common SCHLENK technique [1]. Liquids were transferred via plastic syringes and V2A-steel needles. Powdered solids were added against an argon flow.

Reactions at 0 °C were cooled with an ice/water bath.

Purification by column chromatography was performed according to STILL [2]. Silica gel 60 (MERCK, 0.04–0.063 mm) served as a stationary phase. Solvents for elution were purchased in an analytical-grade purity and used mixtures are reported as volume ratios (v/v).

Freeze-drying of aqueous solutions was performed on a lyophilisator from CHRIST model Alpha 1-2 LD plus, equipped with a VACUUBRAND RZ 2.5 pump.

Solid-phase synthesis

Solid-phase synthesis of linear peptoids was performed by the published submonomer technique [3, 4] in 6 mL plastic-fritted syringes (MULTISYNTHEC GmbH), closed with a plastic cap. As a solid support, a 2-chlorotrityl chloride resin (CARBOLUTION, 1.60 mmol/g loading density) was used. Reaction steps were performed on a KS 501 digital circular shaker (IKA-LABORTECHNIK) at room temperature. Yields were calculated according to the resin loading value.

General Procedure (GP1) for the synthesis of cyclic peptoids [3, 5]

For the synthesis of linear precursors in a fritted syringe, a 2-chlorotrityl chloride resin (300 mg, 0.480 mmol, 1.60 mmol/mg loading density, 100-200 mesh, 1.00 equiv.) was swollen in 3.00 mL of methylene chloride for 2 h. After filtration, a freshly prepared solution of bromoacetic acid (2.59 mmol, 5.40 equiv.) and *N,N'*-diisopropylethylamine (DIPEA, 2.59 mmol, 5.40 equiv.) in 2.5 mL of DCM was added and shaken for 1 h at 21 °C. The resin was extensively washed with peptide grade *N,N'*-dimethyl-formamide (*p*DMF). For the following substitution reaction, a solution of the corresponding amine (3.98 mmol, 8.30 equiv.) in 2.5 mL of *p*DMF was added to the resin and shaken for 30 min at room temperature (overnight in case of aniline). Following extensive washing with *p*DMF, a solution of bromoacetic acid (4.80 mmol, 10.0 equiv.) and *N,N'*-diisopropylcarbodiimide (DIC, 4.80 mmol, 10.0 equiv.) in 2.5 mL *p*DMF were added and shaken for 30 min at room temperature (2 h in the case of aniline). The acetylation and substitution steps were alternated repeatedly until the desired peptoid length was achieved. For cleavage, a solution of 33% hexafluoroisopropanol in DCM was added and the mixture was shaken overnight. The solvent was removed under reduced pressure and the residue was resolved in 10.0 mL of acetonitrile/water (1:1). The mixture was lyophilized overnight to gain a colorless powder.

Without any purification, the full amount of the linear precursor was resolved in 100 mL of dry methylene chloride and degassed with argon. For the cyclization following a protocol by KIRSHENBAUM [5], benzotriazol-1-yl-oxytripyrrolidino-phosphonium hexafluorophosphate (PyBOP, 1.44 mmol, 3.00 equiv.) and DIPEA (2.88 mmol, 6.00 equiv.) were added. The mixture was stirred at room temperature overnight. The solvent was removed under reduced pressure and purification was performed *via* preparative reverse phase HPLC.

General Procedure (GP2) for the CuAAC of two peptoids [6]

Under inert conditions, both peptoids (1.00 equiv. each) were dissolved in dry methylene chloride. Afterwards, 2,6-lutidine (6.00 equiv.) was added to the solution and stirred for 5 min. Then, tetrakis(acetonitrile)copper(I) hexafluorophosphate ($Cu(CH_3CN)_4PF_6$, 1.00 equiv.) was added and the mixture was stirred for 3 days. The solvent was removed under reduced pressure and the product was purified *via* preparative reverse phase HPLC.

General Procedure (GP3) for the CuAAC of a peptoid and a linker [6]

Under inert conditions, the peptoid (1.00 equiv.) and the linker (5.00–10.0 equiv.) were dissolved in dry methylene chloride. Afterwards, 2,6-lutidine (8.00 equiv.) was added to the solution and stirred for 5 min. Then, $Cu(CH_3CN)_4PF_6$ (1.00 equiv.) was added and the mixture was stirred for 3 days. The solvent was removed under reduced pressure and the product was purified *via* preparative reverse phase HPLC.

Synthesis of small molecules

3-Azidopropan-1-amine (17) [7]:

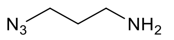

3-Chloropropylamine hydrochloride (6.00 g, 46.0 mmol, 1.00 equiv.) was dissolved in 100 mL of water. Sodium azide (9.00 g, 138 mmol, 3.00 equiv.) was added and the mixture was stirred for 18 h at 21 °C. The solvent was reduced to 2/3 of its volume. The remaining mixture was cooled to 0 °C and 100 mL of diethyl ether were added. Potassium hydroxide (6.00 g, 107 mmol, 2.30 equiv.) was added in portions and the organic layer was separated. The aqueous layer was extracted twice with diethyl ether, the organic layers were combined, dried over Na_2SO_4 and the solvent was removed under reduced pressure. The product was obtained as a colorless liquid in 82% yield (3.78 g, 37.8 mmol).

^{1}H NMR (300 MHz, $CDCl_3$): δ = 1.24 (bs, 2H, N*H*$_2$), 1.67 (quin, *J* = 6.8 Hz, 2H, $CH_2C\mathit{H_2}CH_2$), 2.74 (t, *J* = 6.8 Hz, 2H, C*H*$_2$NH$_2$), 3.31 (t, *J* = 6.8 Hz, 2H, N$_3$C*H*$_2$) ppm. Data are consistent with the literature [7].

4-Azidoaniline (18) [8, 9]:

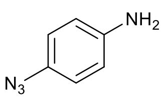

4-Iodoaniline (3.50 g, 16.0 mmol, 1.00 equiv.), sodium azide (1.25 g, 19.0 mmol, 1.20 equiv.), copper(I)iodide (152 mg, 798 µmol, 0.0500 equiv.), L-proline (368 mg, 3.20 mmol, 0.200 equiv.) and sodium hydroxide (128 mg, 3.20 mmol, 0.200 equiv.) was degassed with argon. 40 mL of dry dimethyl sulfoxide were added and the mixture was stirred for 24 h at 40 °C. After cooling to room temperature, 100 mL of water were added and the aqueous layer was extracted with ethyl acetate (3 x 50 mL). The combined organic layers were dried over $MgSO_4$ and the solvent was removed under reduced pressure. The residue was purified *via* flash column chromatography on silica gel (methylene chloride). The product was obtained as brown crystals in 62% yield (1.32 g, 9.84 mmol).

^{1}H NMR (300 MHz, $CDCl_3$): δ = 3.64 (bs, 2H, N*H*$_2$), 6.67(d, *J* = 8.8 Hz, 2H, 2 × C*H*$_{Ar}$), 6.84 (d, *J* = 8.8 Hz, 2H, 2 × C*H*$_{Ar}$) ppm. – **IR** (ATR): $\tilde{\upsilon}$ = 3393 (m), 3320 (m), 3225 (m), 2421 (w), 2254 (w), 2102 (m), 2068 (m), 1631 (m), 1600 (m), 1500 (m), 1302 (m), 1266 (m), 1126 (m), 1082 (m), 833 (m), 815 (m), 783 (m), 623 (m), 507 (s), 438 (m), 414 (m). – **MS** (EI, 70 eV): *m*/*z* (%) = 134 (71) $[M]^+$, 106 (100) $[M\text{-}N_2]^+$. Data are consistent with the literature [9].

1,2-Bis(4-bromophenyl)diazene (19) [10]:

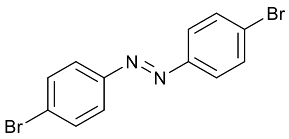

40 mL of aqueous potassium peroxymonosulfate (5.28 g, 8.60 mmol, 2.00 equiv.) were added dropwise to a solution of 4-bromoaniline (740 mg, 4.30 mmol, 1.00 equiv.) in 10 mL methylene chloride. The reaction mixture was stirred for 2 h at 21 °C. The organic layer was separated, repeatedly washed with water and dried over $MgSO_4$. The solvent was removed under reduced pressure and the residue was dissolved in 50 mL of a mixture of glacial acetic acid and ethyl acetate (1:1). 4-Bromoaniline (592 mg, 3.40 mmol, 0.800 equiv.) was added and the mixture was stirred overnight at 40°C. During cooling to room temperature, an orange solid precipitated. The solid was filtered off and repeatedly washed with ethyl acetate. 1,2-Bis(4-

bromophenyl)diazene was obtained as an orange solid in 45% yield (663 mg, 1.96 mmol).

^{1}H NMR (300 MHz, $CDCl_3$): δ = 7.63(d, *J* = 9.0 Hz, 4H, 4 × C*H*Ar), 8.04 (d, *J* = 9.0 Hz, 4H, 4 × C*H*Ar) ppm. – **IR** (ATR): $\tilde{\upsilon}$= 2574 (vw), 2136 (vw), 1904 (vw), 1566 (vw), 1486 (vw), 1469 (vw), 1396 (vw), 1278 (vw), 1096 (w), 1062 (w), 1003 (w), 832 (w), 814 (w), 709 (vw), 605 (vw), 535 (w), 489 (vw), 407 (vw). – **MS** (EI, 70 eV): *m*/*z* (%) = 340 (71) $[M]^+$, 183 (70) $[M\text{-}Ar\text{-}Br]^+$, 155 (100) $[M\text{-}N_2\text{-}Ar\text{-}Br]^+$. Data are consistent with the literature [10].

1,2-Bis(4-((trimethylsilyl)ethynyl)phenyl)diazene (20) [11]:

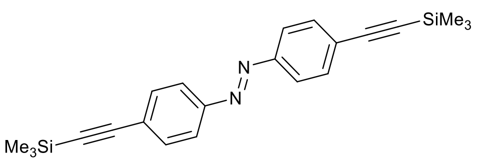

1,2-Bis(4-bromophenyl)diazene (**19**, 510 mg, 1.50 mmol, 1.00 equiv.), copper(I)-iodide (28.5 mg, 0.150 mmol, 0.100 equiv.), triphenylphosphine (63.0 mg, 0.240 mmol, 0.160 equiv.) and tris(dibenzylideneacetone)-dipalladium(0) (27.5 mg, 0.0300 mmol, 0.0200 equiv.) were mixed and degassed. After the addition of 10 mL of dry trimethylamine, the reaction was stirred for 10 min. Trimethylsilylacetylene (368 mg, 3.75 mmol, 2.50 equiv.) was added and the reaction was stirred for 5 days at 90 °C. The solvent was removed and the residue was dissolved in diethyl ether. The organic layer was washed with water, dried over $MgSO_4$ and the solvent was removed under reduced pressure. After purification *via* flash chromatography on silica gel (cyclohexane/methylene chloride = 3:1) the product was obtained as an orange solid in 90% yield (505 mg, 1.35 mmol).

^{1}H NMR (300 MHz, $CDCl_3$): δ = 0.27 (s, 18H, 6 × C*H*$_3$), 7.60 (d, *J* = 8.5 Hz, 4H, 4 × C*H*$_{Ar}$), 7.86 (d, *J* = 8.5 Hz, 4H, 4 × C*H*$_{Ar}$) ppm. – **MS** (EI, 70 eV): *m*/*z* (%) = 374 (100) $[M]^+$, 173 (50) $[M\text{-}Ar\text{-}CCSi(CH_3)_3]^+$, 158 (11) $[M\text{-}N_2\text{-}Ar\text{-}CCSi(CH_3)_3]^+$. Data are consistent with the literature [11].

1,2-Bis(4-ethynylphenyl)diazene (21) [12]:

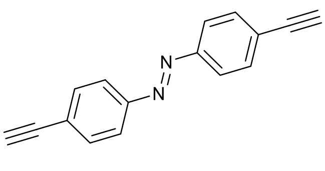

Under inert conditions, 1,2-bis(4-((trimethylsilyl)ethynyl)-phenyl)diazene (**20**, 340 mg, 1.00 mmol, 1.00 equiv.) was dissolved in 15 mL of dry methanol. After the addition of potassium carbonate (163 mg, 1.18 mmol, 1.30 equiv.), the mixture was stirred for 24 h at 21 °C. The precipitated solid was filtered off and washed with water. The product was obtained as an orange-brown solid in 99% yield (207 mg, 0.900 mmol).

^{1}H NMR (300 MHz, $CDCl_3$): δ = 3.24 (s, 2H, 2 × C*H*), 7.64 (d, *J* = 8.5 Hz, 4H, 4 × C*H*$_{Ar}$), 7.89 (d, *J* = 8.5 Hz, 4H, 4 × C*H*$_{Ar}$) ppm. – **IR** (ATR): υ= 3258 (s), 1637 (w), 1489 (w), 1406 (w), 1229 (w), 1154 (w), 1101 (w), 1008 (vw), 844 (m), 733 (w), 704 (w), 675 (m), 634 (m), 555 (m), 487 (m). – **MS** (EI, 70 eV): *m*/*z* (%) = 230 (100) $[M]^+$, 129 (23) $[M\text{-}Ar\text{-}CCH]^+$, 101 (54) $[M\text{-}N_2\text{-}Ar\text{-}CCH]^+$. Data are consistent with the literature [12].

4,4'-(diazene-1,2-diyl)diphenol (22) [13, 14]:

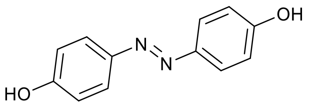

Aminophenol (2.20 g, 20.1 mmol, 1.00 equiv.) was dissolved in 8.50 mL of 5 M HCl (50.0 mmol, 2.50 equiv.) and stirred for 45 min at 21 °C. After cooling to 0–5 °C, sodium nitrite (1.39 g, 20.1 mmol, 1.00 equiv.) was dissolved in 9.00 mL of water and added dropwise to the reaction. After 2 h, phenol (1.52 g, 16.1 mmol, 0.800 equiv.) was dissolved in 18.5 mL of 2 M NaOH and was added slowly to the aminophenol solution, not exceeding a temperature of 5 °C. The reaction was neutralized with Na_2CO_3 and stirred for another 10 h at 21 °C. During the subsequent dropwise addition of 1 M HCl, a red solid precipitated. The solid was filtered off and washed with water. The product was obtained as a red solid in 35% yield (2.68 g, 12.5 mmol).

^{1}H NMR (300 MHz, $CDCl_3$): δ = 6.89 (d, *J* = 9.1 Hz, 4H, 4 × C*H*$_{Ar}$), 7.88 (d, *J* = 9.1 Hz, 4H, 4 × C*H*$_{Ar}$) ppm. – **IR** (ATR): υ = 3187 (vw), 2132 (vw), 1658 (vw), 1588 (w), 1503 (vw), 1475 (w), 1427 (vw), 1378(vw), 1247 (w), 1151 (w), 1103 (w), 830 (w), 771 (vw), 712 (vw), 646 (vw), 531 (vw), 470 (vw), 432 (vw), 414 (vw). – **MS** (EI, 70 eV): *m*/*z* (%) = 214 (100) $[M]^+$, 121 (25) $[M\text{-}Ar\text{-}OH]^+$, 93 (29) $[M\text{-}N_2\text{-}Ar\text{-}OH]^+$. Data are consistent with the literature [14].

1,2-Bis(4-(prop-2-yn-1-yloxy)phenyl)diazene (23) [15]:

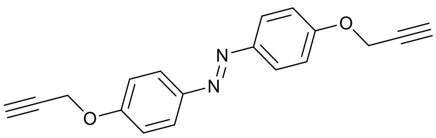

Under inert conditions, 4,4'-(diazene-1,2-diyl)diphenol (**22**, 500 mg, 2.33 mmol, 1.00 equiv.) and potassium carbonate (1.60 g, 11.6 mmol, 5.00 equiv.) was dissolved in 30 mL of dry acetone and stirred for 30 min at 21 °C. After addition of propargyl bromide (1.67 g, 14.0 mmol, 6.00 equiv.), the mixture was stirred for another 24 h at 21 °C. The solvent was removed under reduced pressure and the compound was purified *via* column chromatography (diethyl ether). The product was obtained as a red solid in 89% yield (606 mg, 2.09 mmol). Compound **18** was synthesized as described in the literature.[14] Under inert conditions 500 mg azophenole (2.33 mmol, 1.00 equiv.) and 1.60 g K_2CO_3 (11.6 mmol, 5.00 equiv.) were dissolved in 30 mL dry acetone and stirred for 30 min. After addition of 1.70 g propargyl bromide (14.0 mmol, 6.00 equiv.) the mixture was stirred at room temperature for another 24 h. The solvent was removed under reduced pressure and purified *via* flash column chromatography on silica gel (diethyl ether). The product was obtained as a red solid in 90% yield (606 mg, 2.09 mmol).

^{1}H NMR (300 MHz, $CDCl_3$): δ = 3.64 (s, 2H, 2 × C*H*), 3.64 (s, 4H, 2 × C*H*$_2$), 6.67 (d, ^3J = 8.8 Hz, 4H, 4 × C*H*$_{Ar}$), 6.84 (d, ^3J = 8.8 Hz, 4H, 4 × C*H*$_{Ar}$) ppm. – **IR** (ATR): υ = 3274 (w), 2129 (vw), 1593 (m), 1497 (m), 1451 (w), 1423 (w), 1377 (w), 1296 (w), 1232 (m), 1146 (m), 1107 (w), 1015 (m), 973 (w), 842 (m), 808 (w), 783 (w), 730 (vw), 709 (w), 666 (m), 555 (m), 530 (w), 461 (w). – **MS** (EI, 70 eV): *m*/*z* (%): 290 (100) $[M]^+$, 251 (43) $[M\text{-}CH_2CCH]^+$, 159 (19) $[M\text{-}Ar\text{-}O\text{-}CH_2CCH]^+$, 131 (27) $[M\text{-}N_2\text{-}Ar\text{-}O\text{-}CH_2CCH]^+$. Data are consistent with the literature [15].

Synthesis of macrocyclic peptoids

c-[(Nph)$_2$N2moN1ay]$_2$ (7a) [16]:

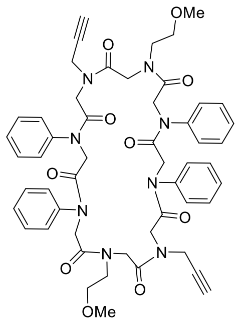

Peptoid **7a** was synthesized following **GP1**. Linkage to the resin was carried out using bromoacetic acid (360 mg, 2.59 mmol, 5.40 equiv.) and DIPEA (463 µL, 343 mg, 2.59 mmol, 5.40 equiv.) in 2.5 mL of methylene chloride. The following acylation steps were carried out using bromoacetic acid (667 mg, 4.80 mmol, 10.0 equiv.) and DIC (721 µL, 581 mg, 4.80 mmol, 10.0 equiv.) in 3.00 mL of *p*DMF. For the substitution steps, aniline (*N*ph, 364 µL, 371 mg, 3.98 mmol, 8.30 equiv.), 2-methoxyethylamine (*N*2mo, 348 µL, 301 mg, 3.98 mmol, 8.30 equiv.) and propargylamine (*N*1ay, 255 µL, 219 mg, 3.98 mmol, 8.30 equiv.) in 3.00 mL of *p*DMF were added, respectively. The product formation of the linear precursor was confirmed *via* MALDI-TOF-MS.

MS (MALDI-TOF, DHB/CHCA 1:1): *m/z* = 971 $[M+H]^+$.

The cyclization of the linear peptoid was performed using PyBOP (748 mg, 1.44 mmol, 3.00 equiv.) and DIPEA (490 µL, 264 mg, 2.88 mmol, 6.00 equiv.). After purification, *via* preparative reverse phase HPLC (5–95% acetonitrile in water with 0.1% TFA over 30 min) the product was obtained as a colorless solid in 11% yield (50.0 mg, 52.5 µmol).

MS (MALDI-TOF, DHB/CHCA 1:1): *m/z* = 953 $[M+H]^+$. – **Analytical HPLC** (5–95% acetonitrile in water with 0.1% TFA over 30 min): t_{ret} = 17.1 min (99%).

c-[(Nph)$_2$N2moN3az]$_2$ (7b):

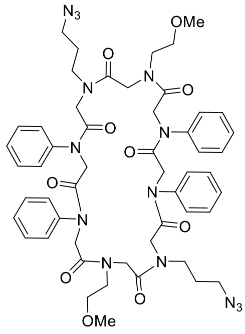

Peptoid **7b** was synthesized following **GP1**. Linkage to the resin was carried out using bromoacetic acid (360 mg, 2.59 mmol, 5.40 equiv.) and DIPEA (463 µL, 343 mg, 2.59 mmol, 5.40 equiv.) in 2.5 mL of methylene chloride. The following acylation steps were carried out using bromoacetic acid (667 mg, 4.80 mmol, 10.0 equiv.) and DIC (721 µL, 581 mg, 4.80 mmol, 10.0 equiv.) in 3.00 mL of *p*DMF. For the substitution steps, aniline (*N*ph, 364 µL, 371 mg, 3.98 mmol, 8.30 equiv.), 2-methoxyethylamine (*N*2mo, 348 µL, 301 mg, 3.98 mmol, 8.30 equiv.) and 3-azidopropylamine (*N*3az, 398 mg, 3.98 mmol, 8.30 equiv.) in 3.00 mL of *p*DMF were added, respectively. The product formation of the linear precursor was confirmed *via* MALDI-TOF-MS.

MS (MALDI-TOF, DHB/CHCA 1:1): *m/z* = 1061 $[M+H]^+$.

The cyclization of the linear peptoid was performed using PyBOP (748 mg, 1.44 mmol, 3.00 equiv.) and DIPEA (490 µL, 264 mg, 2.88 mmol, 6.00 equiv.). After purification, *via* preparative reverse phase HPLC (5–95% acetonitrile in water with 0.1% TFA over 30 min) the product was obtained as a colorless solid in 25% yield (123 mg, 0.118 mmol). **MS** (MALDI-TOF, DHB/CHCA 1:1): *m/z* = 1043 $[M+H]^+$. – **Analytical HPLC** (5–95% acetonitrile in water with 0.1% TFA over 30 min): t_{ret} =16.5 min (98%).

c-[*N*ph*N*ph^{p}ay(*N*2mo)$_2$]$_2$ (7c):

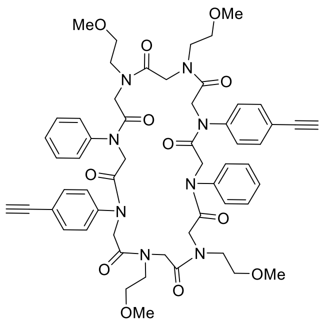

Peptoid **7c** was synthesized following **GP1**. Linkage to the resin was carried out using bromoacetic acid (360 mg, 2.59 mmol, 5.40 equiv.) and DIPEA (463 µL, 343 mg, 2.59 mmol, 5.40 equiv.) in 2.5 mL of methylene chloride. The following acylation steps were carried out using bromoacetic acid (667 mg, 4.80 mmol, 10.0 equiv.) and DIC (721 µL, 581 mg, 4.80 mmol, 10.0 equiv.) in 3.00 mL of *p*DMF. For the substitution steps aniline (*N*ph, 364 µL, 371 mg, 3.98 mmol, 8.30 equiv.), 4-ethynylaniline (*N*ph^{p}ay, 466 mg, 3.98 mmol, 8.30 equiv.) and 2-methoxyethylamine (*N*2mo, 348 µL, 301 mg, 3.98 mmol, 8.30 equiv.) in 3.00 mL of *p*DMF were added, respectively. The product formation of the linear precursor was confirmed *via* MALDI-TOF-MS.

MS (MALDI-TOF, DHB/CHCA 1:1): m/z = 1059 $[M+H]^+$.

The cyclization of the linear peptoid was performed using PyBOP (748 mg, 1.44 mmol, 3.00 equiv.) and DIPEA (490 µL, 264 mg, 2.88 mmol, 6.00 equiv.). After purification, *via* preparative reverse phase HPLC (5–95% acetonitrile in water with 0.1% TFA over 30 min) the product was obtained as a colorless solid in 8% yield (40.0 mg, 38.4 µmol).

MS (MALDI-TOF, DHB/CHCA 1:1): m/z = 1041 $[M+H]^+$. – **Analytical HPLC** (5–95% acetonitrile in water with 0.1% TFA over 30 min): t_{ret} =16.0 min (96%).

c-[*N*ph*N*ph^{p}az(*N*2mo)$_2$]$_2$ (7d):

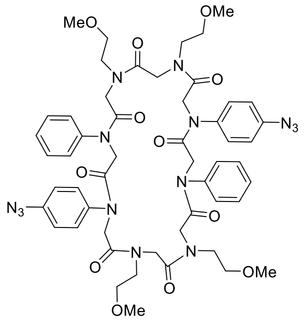

Peptoid **7d** was synthesized following **GP1**. Linkage to the resin was carried out using bromoacetic acid (360 mg, 2.59 mmol, 5.40 equiv.) and DIPEA (463 µL, 343 mg, 2.59 mmol, 5.40 equiv.) in 2.5 mL of methylene chloride. The following acylation steps were carried out using bromoacetic acid (667 mg, 4.80 mmol, 10.0 equiv.) and DIC (721 µL, 581 mg, 4.80 mmol, 10.0 equiv.) in 3.00 mL of *p*DMF. For the substitution steps aniline (*N*ph, 364 µL, 371 mg, 3.98 mmol, 8.30 equiv.), 4-azidoaniline (*N*ph^{p}az, 533 mg, 3.98 mmol, 8.30 equiv.) and 2-methoxyethylamine (*N*2mo, 348 µL, 301 mg, 3.98 mmol, 8.30 equiv.) in 3.00 mL of *p*DMF were added, respectively. The product formation of the linear precursor was confirmed *via* MALDI-TOF-MS.

MS (MALDI-TOF, DHB/CHCA 1:1): m/z = 1093 $[M+H]^+$.

The cyclization of the linear peptoid was performed using PyBOP (748 mg, 1.44 mmol, 3.00 equiv.) and DIPEA (490 µL, 264 mg, 2.88 mmol, 6.00 equiv.). After purification, *via* preparative reverse phase HPLC (5–95% acetonitrile in water with 0.1% TFA over 30 min) the product was obtained as a colorless solid in 9% yield (48.0 mg, 44.6 µmol).

MS (MALDI-TOF, DHB/CHCA 1:1): m/z = 1075 $[M+H]^+$. – **Analytical HPLC** (5–95% acetonitrile in water with 0.1% TFA over 30 min): t_{ret} =16.2 min (87%).

Combination-[*c*-[(*N*ph)$_2$*N*2mo*N*1ay]$_2$ - *c*-[(*N*ph)$_2$*N*2mo*N*3az] (8):

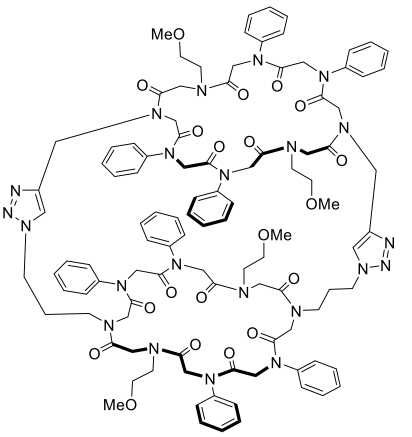

The 1,3-dipolar cycloaddition was performed following **GP2**. Peptoid **7a** (18.6 mg, 19.1 µmol, 1.00 equiv.) and peptoid **7b** (20.0 mg, 19.1 µmol, 1.00 equiv.) were dissolved in 30 mL of methylene chloride and degassed with argon. 2,6-Lutidine (14.0 µL, 13.0 mg, 115 µmol, 6.00 equiv.) and Cu(CH$_3$CN)$_4$PF$_6$ (7.20 mg, 19.1 µmol, 1.00 equiv.) were added stepwise. After filtration, the product was purified *via* preparative reverse phase HPLC (30–100% acetonitrile in water with 0.1% TFA over 30 min) to obtain a colorless powder in 7% yield (2.50 mg, 1.25 µmol).

MS (MALDI-TOF, DHB/CHCA 1:1): m/z = 1997 [M+1]$^+$ – **Analytical HPLC** (5–95% acetonitrile in water with 0.1% TFA over 30 min): t_{ret} = 17.7 min (99%).

Combination-[*c*-[*N*ph*N*ph^{p}ay(*N*2mo)$_2$]$_2$ - *c*-[*N*ph*N*ph^{p}az(*N*2mo)$_2$]$_2$] (9):

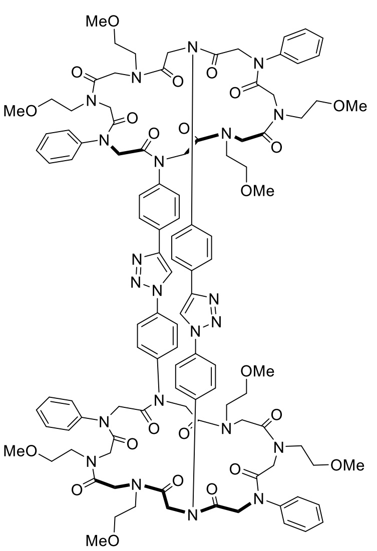

The 1,3-dipolar cycloaddition was performed following **GP2**. Peptoid **7c** (19.3 mg, 18.6 µmol, 1.00 equiv.) and peptoid **7d** (20.0 mg, 18.6 µmol, 1.00 equiv.) were dissolved in 30 mL of methylene chloride and degassed with argon. 2,6-Lutidine (14.0 µL, 13.0 mg, 115 µmol, 6.00 equiv.) and Cu(CH$_3$CN)$_4$PF$_6$ (7.20 mg, 19.1 µmol, 1.00 equiv.) were added stepwise. After filtration, the product was purified *via* preparative HPLC (30–100% acetonitrile in water with 0.1% TFA over 30 min) to obtain a colorless powder in 6% yield (2.50 mg, 1.18 µmol).

MS (MALDI-TOF, DHB/CHCA 1:1): m/z = 2117 [M+H]$^+$. – **Analytical HPLC** (5–95% acetonitrile in water with 0.1% TFA over 30 min): t_{ret} = 17.9 min (98%).

Combination-[*c*-[(*N*ph)$_2$*N*2mo*N*3az]$_2$ – (1,4-diethinylbenzene)$_2$] (11):

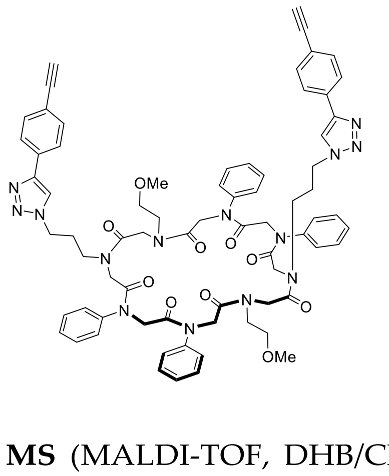

The 1,3-dipolar cycloaddition was performed following **GP3**. Peptoid **7b** (20.0 mg, 19.1 µmol, 1.00 equiv.) and 1,4-diethynylbenzene (12.1 mg, 191 µmol, 10.0 equiv.) were dissolved in 30 mL of methylene chloride and degassed with argon. 2,6-Lutidine (18 µL, 16.7 mg, 153 µmol, 8.00 equiv.) and Cu(CH$_3$CN)$_4$PF$_6$ (7.20 mg, 19.1 µmol, 1.00 equiv.) were added stepwise. After filtration, the product was purified *via* preparative reverse phase HPLC (30–100% acetonitrile in water with 0.1% TFA over 30 min) and the product was obtained as a colorless powder in 17% yield (4.30 mg, 3.32 µmol).

MS (MALDI-TOF, DHB/CHCA 1:1): m/z = 1296 [M+H]$^+$. – **Analytical HPLC** (5–95% acetonitrile in water with 0.1% TFA over 30 min): t_{ret} = 19.4 min (99%).

Combination-[*c*-[(*N*ph)$_2$*N*2mo*N*3az]$_2$ – (1,2-bis(4-(prop-2-yn-1-yloxy)phenyl)diazene)$_2$] (13):

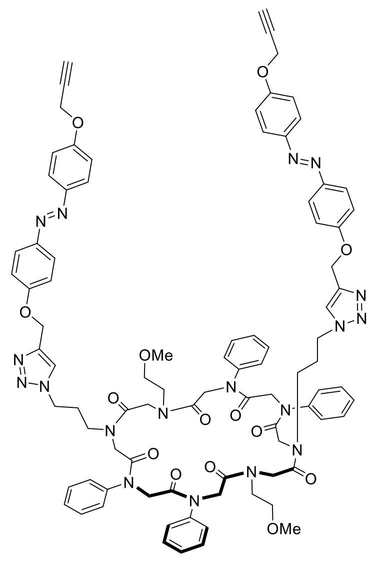

The 1,3-dipolar cycloaddition was performed following **GP3**. Peptoid **7b** (20.0 mg, 19.1 µmol, 1.00 equiv.) and linker **23** (27.8 mg, 95.5 µmol, 5.00 equiv.) were dissolved in 30 mL of methylene chloride and degassed with argon. 2,6-Lutidine (18 µL, 16.7 mg, 153 µmol, 8.00 equiv.) and $Cu(CH_3CN)_4PF_6$ (7.20 mg, 19.1 µmol, 1.00 equiv.) were added stepwise. After filtration, the product was purified *via* preparative reverse phase HPLC (30–100% acetonitrile in water with 0.1% TFA over 30 min) to obtain a colorless powder in 19% yield (6.00 mg, 3.70 µmol).

MS (MALDI-TOF, DHB/CHCA 1:1): *m*/*z* = 1624 $[M+H]^+$. – **Analytical HPLC** (5–95% acetonitrile in water with 0.1% TFA over 30 min): t_{ret} = 14.8 min (98%).

Combination-[*c*-[(*N*ph)$_2$*N*2mo*N*3az]$_2$ – (1,2-bis(4-ethynylphenyl)diazene)$_2$] (15)

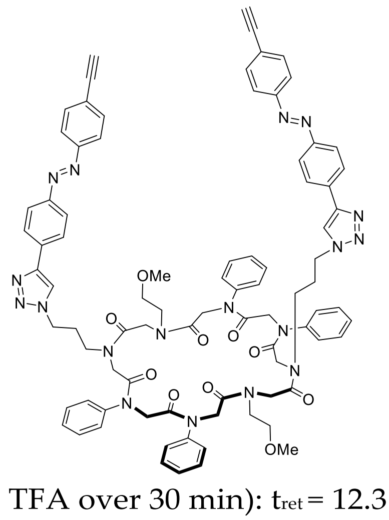

The 1,3-dipolar cycloaddition was performed following **GP3**. Peptoid **7b** (20.0 mg, 19.1 µmol, 1.00 equiv.) and linker **21** (21.9 mg, 95.5 µmol, 5.00 equiv.) were dissolved in 30 mL of methylene chloride and degassed with argon. 2,6-Lutidine (18 µL, 16.7 mg, 152.8 µmol, 8.00 equiv.) and $Cu(CH_3CN)_4PF_6$ (7.20 mg, 19.1 µmol, 1.00 equiv.) were added stepwise. After filtration, the product was purified *via* preparative reverse phase HPLC (30–100% acetonitrile in water with 0.1% TFA over 30 min) to obtain a colorless powder in 3% yield (1.00 mg, 0.666 µmol).

MS (MALDI-TOF, DHB/CHCA 1:1): *m*/*z* = 1503 $[M+H]^+$. – **Analytical HPLC** (5–95% acetonitrile in water with 0.1% TFA over 30 min): t_{ret} = 12.3 min (98%).

Combination-[*c*-[(*N*ph)$_2$*N*2mo*N*3az]$_2$ – (1,2-bis(4-ethynylphenyl)diazene)$_2$ – c-[(*N*ph)$_2$*N*2mo*N*3az]$_2$] (16):

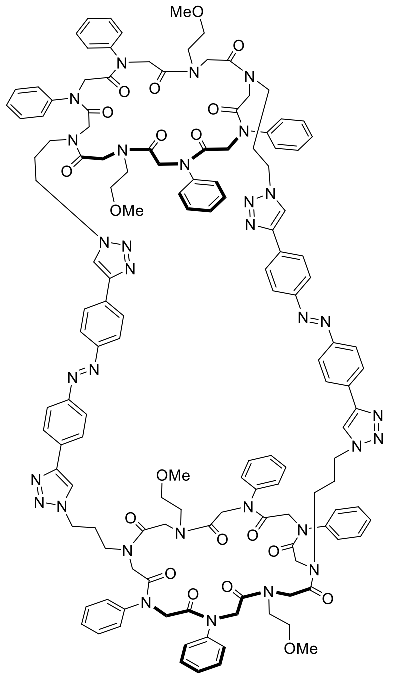

The 1,3-dipolar cycloaddition was performed following **GP3**. peptoid **7b** (20.0 mg, 19.1 µmol, 1.00 equiv.) and linker **21** (21.9 mg, 95.5 mmol, 5.00 equiv.) were dissolved in 30 mL of methylene chloride and degassed with argon. 2,6-Lutidine (18 µL, 16.7 mg, 152.8 µmol, 8.00 equiv.) and $Cu(CH_3CN)_4PF_6$ (7.20 mg, 19.1 µmol, 1.00 equiv.) were added stepwise. After filtration, the product was purified *via* preparative reverse phase HPLC (30–100% acetonitrile in water with 0.1% TFA over 30 min) to obtain a colorless powder in 2% yield (800 µg, 0.313 µmol).

MS (MALDI-TOF, DHB/CHCA 1:1): m/z = 2560 $[M+H]^+$. – **Analytical HPLC** (5–95% acetonitrile in water with 0.1% TFA over 30 min): t_{ret} = 15.6 min (99%).

Further Analytics

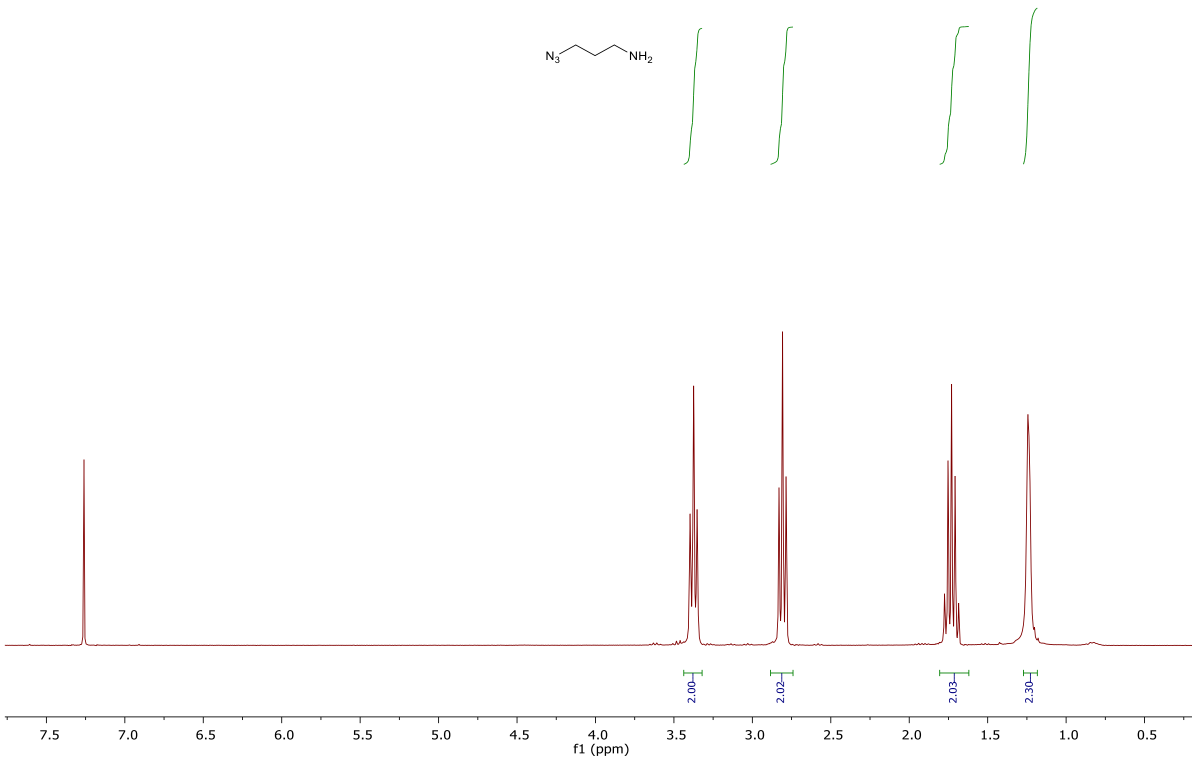

Figure S1: ^{1}H NMR of compound **17**.

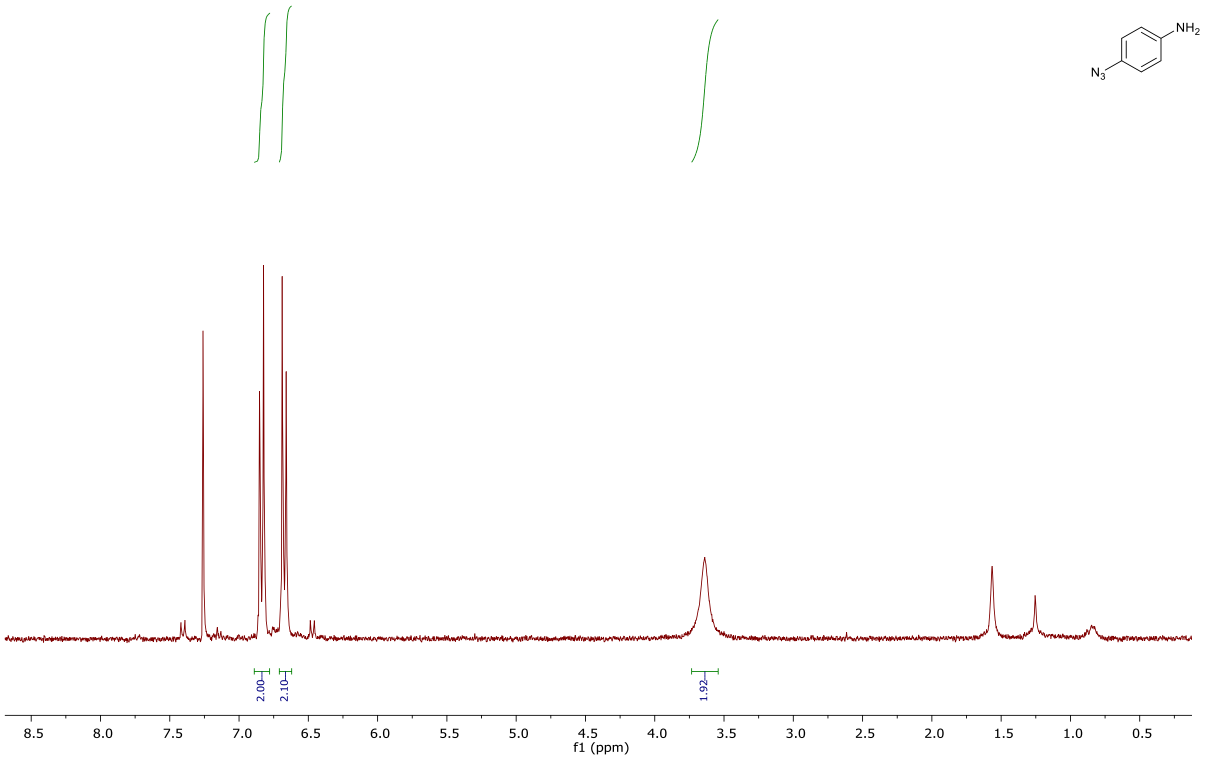

Figure S2: ^{1}H NMR of compound **18**.

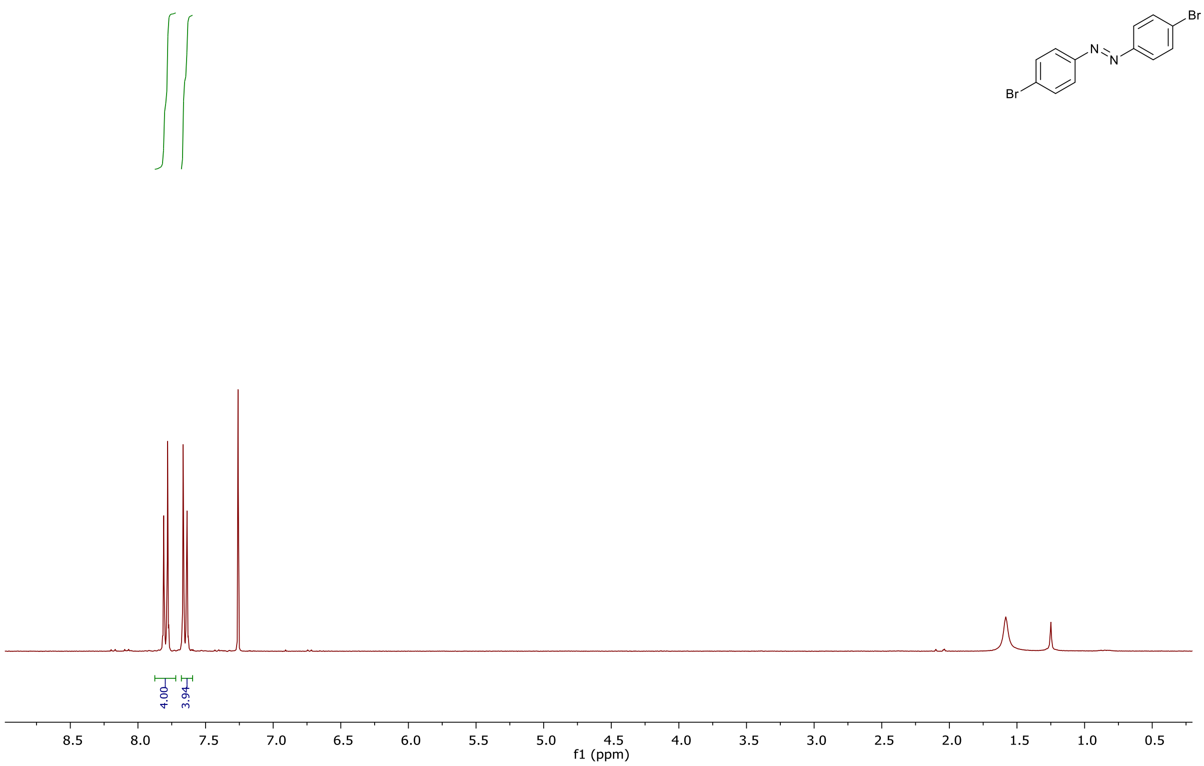

Figure S3: ^{1}H NMR of compound **19.**

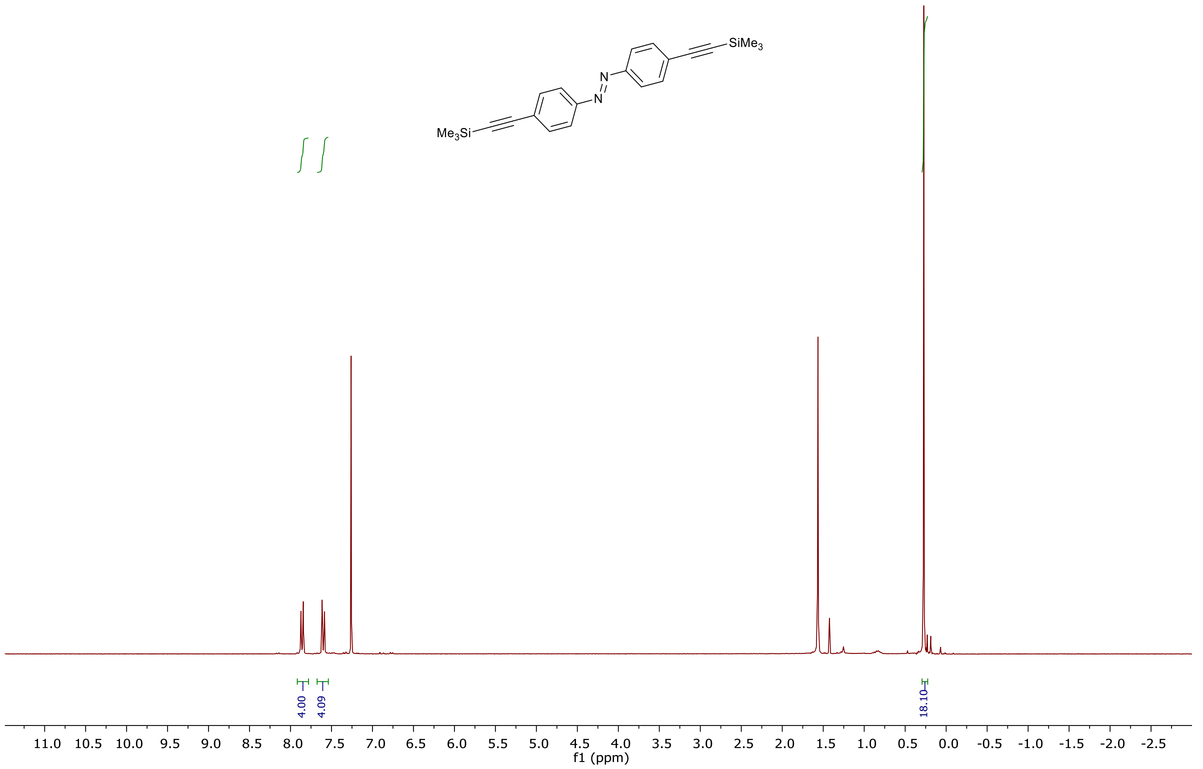

Figure S4: ^{1}H NMR of compound **20.**

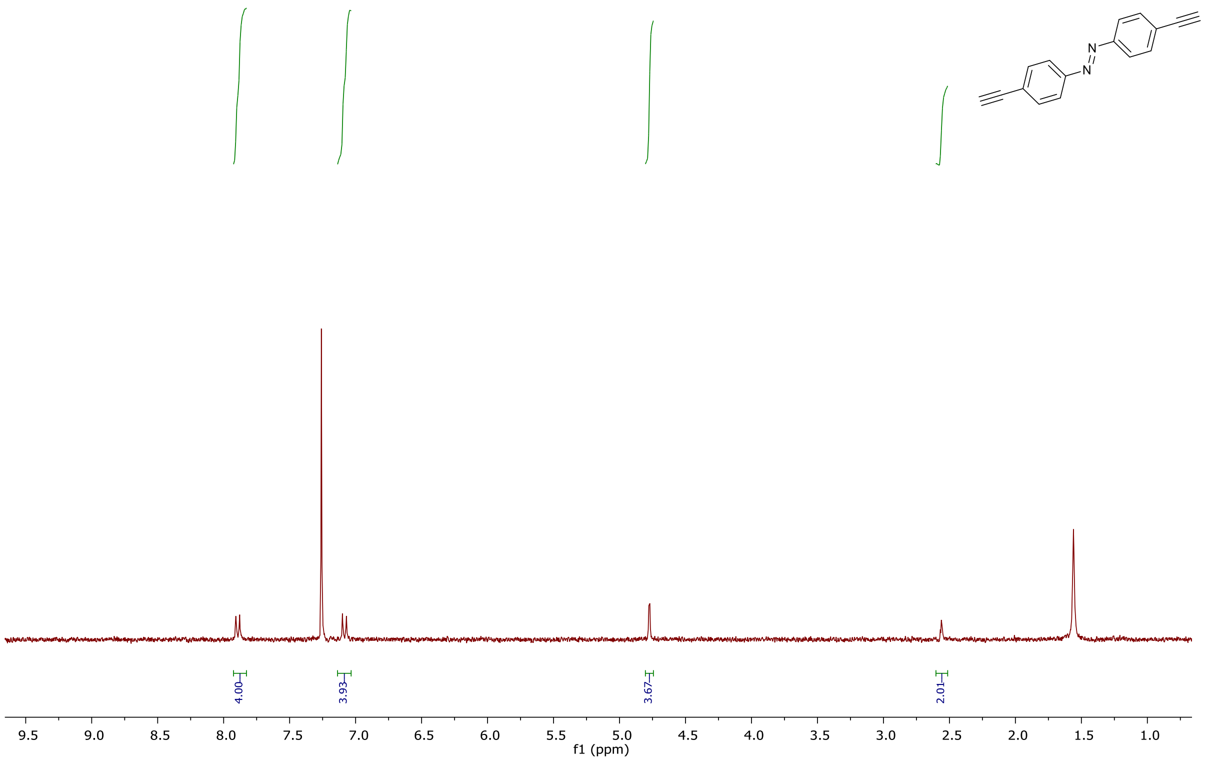

Figure S5: ^{1}H NMR of compound **21.**

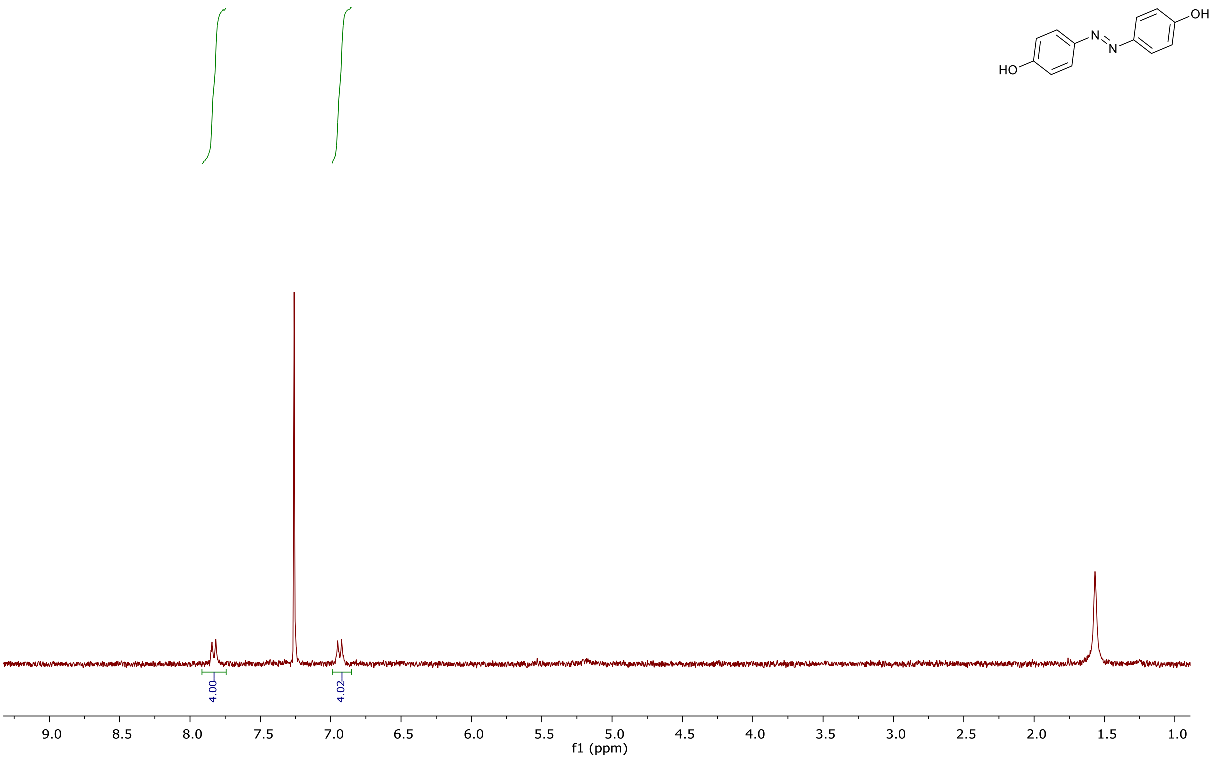

Figure S6: ^{1}H NMR of compound **22.**

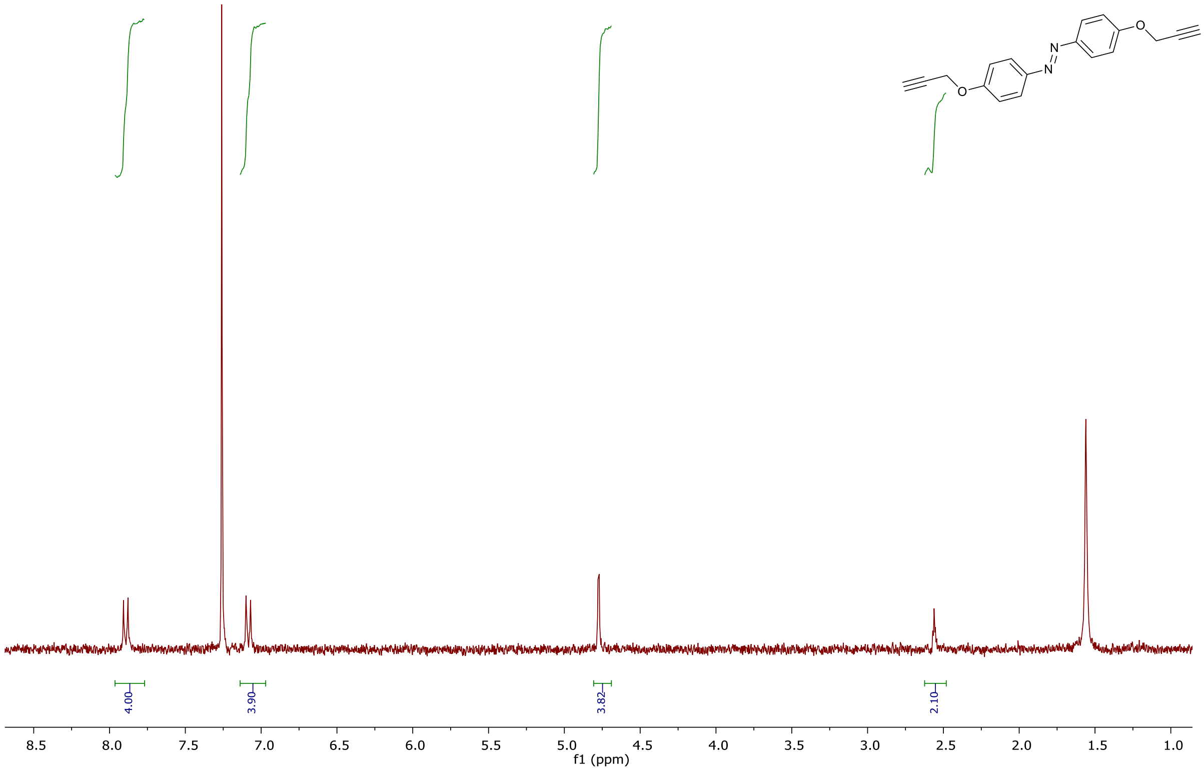

Figure S7: ^{1}H NMR of compound **23.**

Table S1: Analytical HPLC traces of macrocyclic peptoids.

Compound structure	HPLC trace
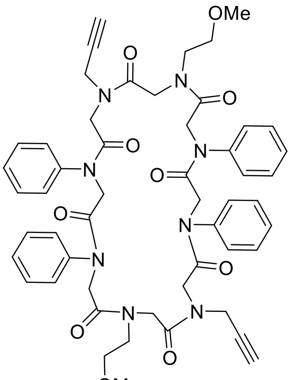 **7a**	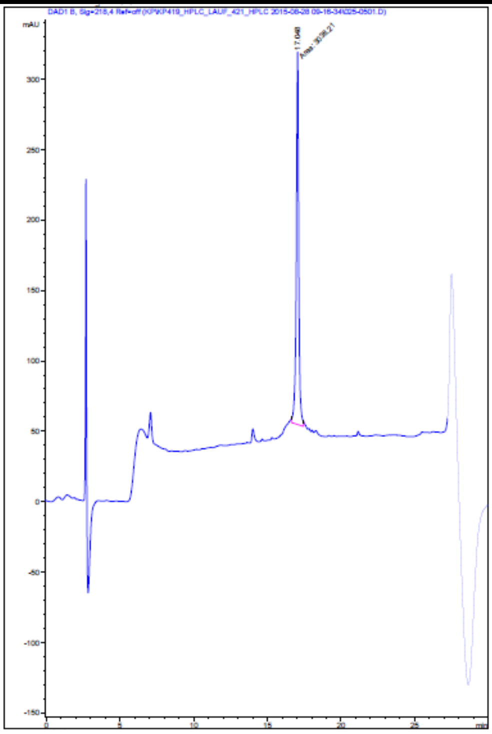
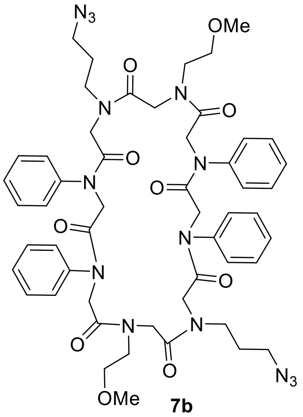 **7b**	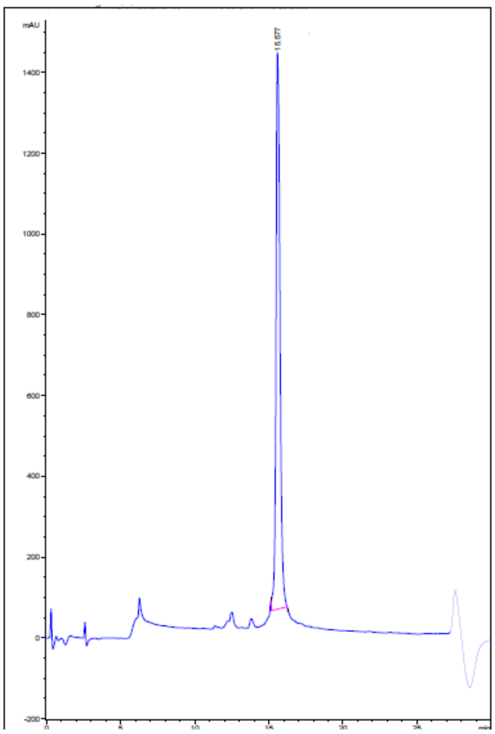

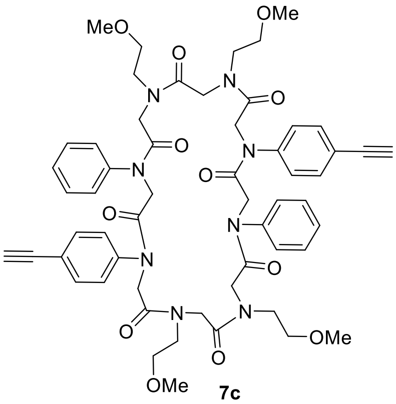
MeO
OMe
OMe
OMe
N
O

7c

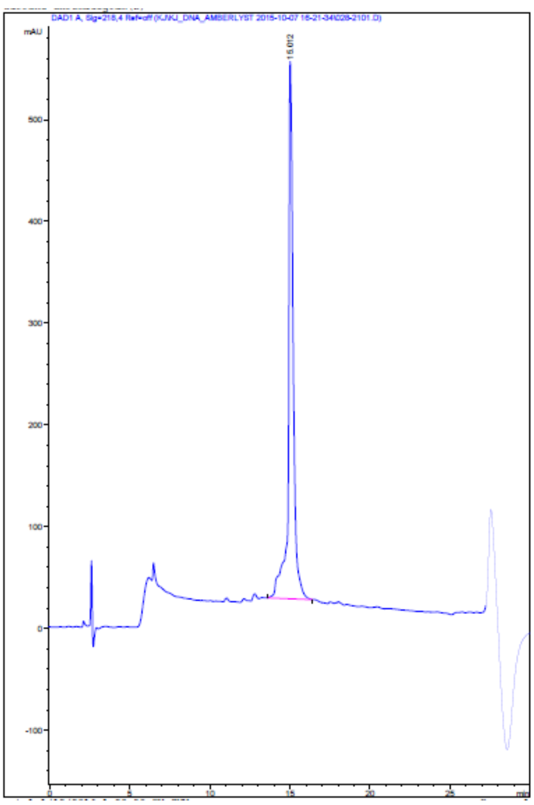
mAU
15.012
500
400
300
200
100
0
-100
0
5
10
15
20
25
min

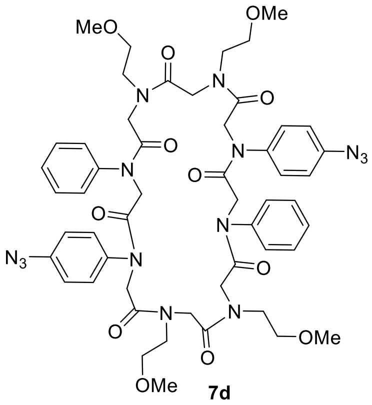
MeO
OMe
OMe
OMe
N
O
N3

7d

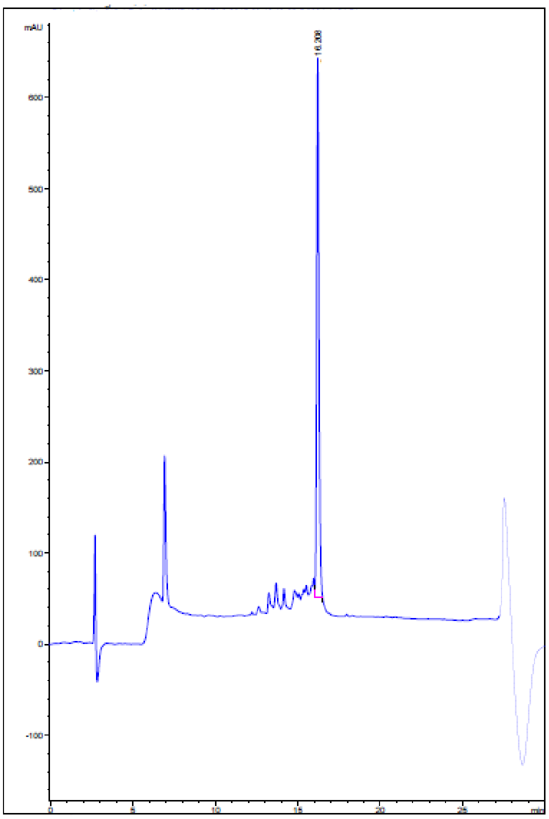
mAU
16.208
600
500
400
300
200
100
0
-100
0
5
10
15
20
25
min

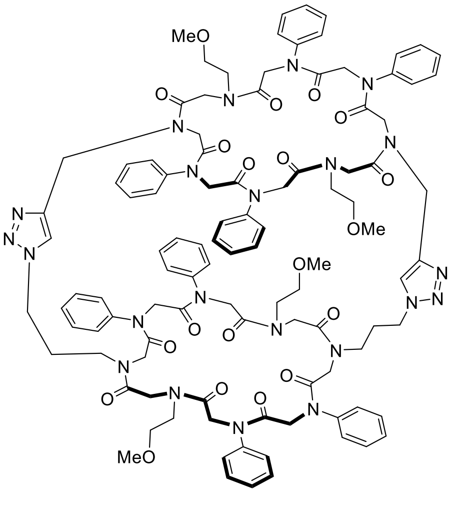

8

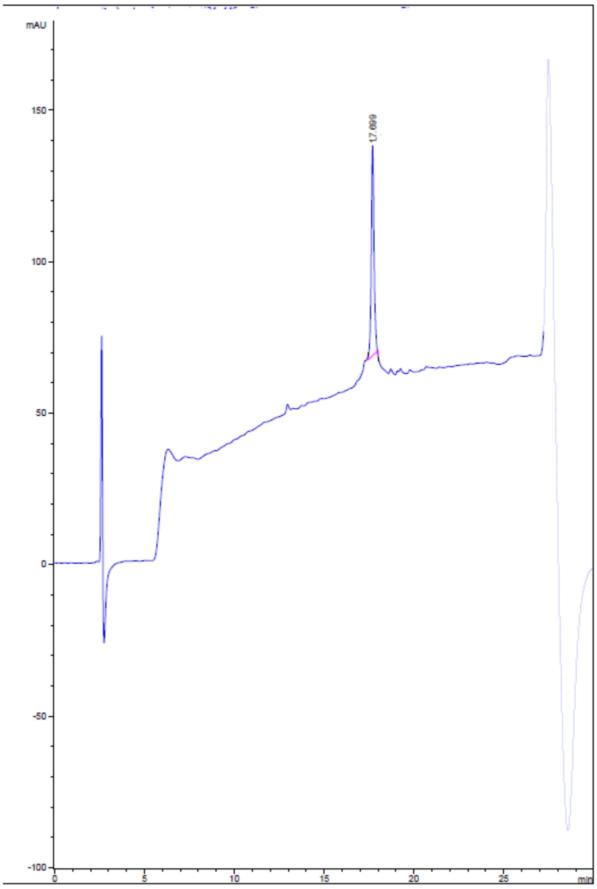

mAU
150
100
50
0
-50
-100
17.699
0
5
10
15
20
25
min

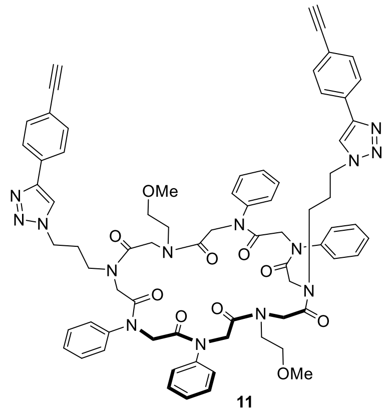

11

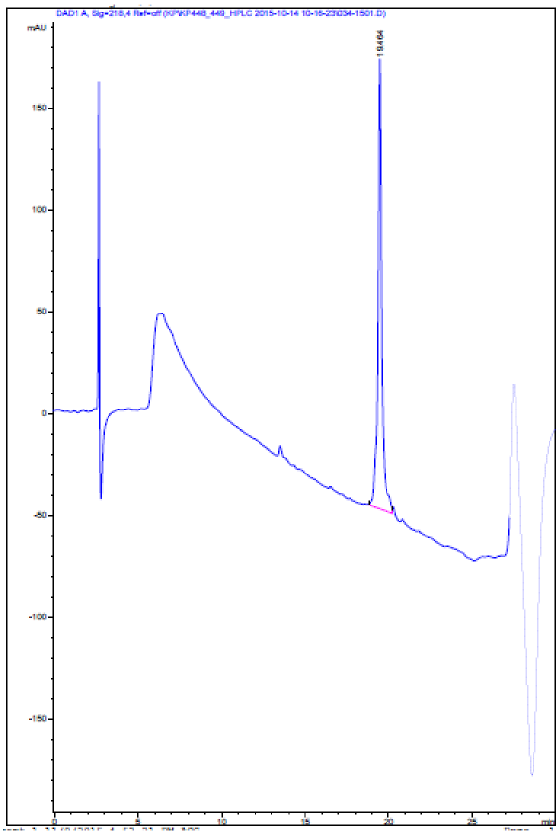

mAU
150
100
50
0
-50
-100
-150
19.464
5
10
15
20
25

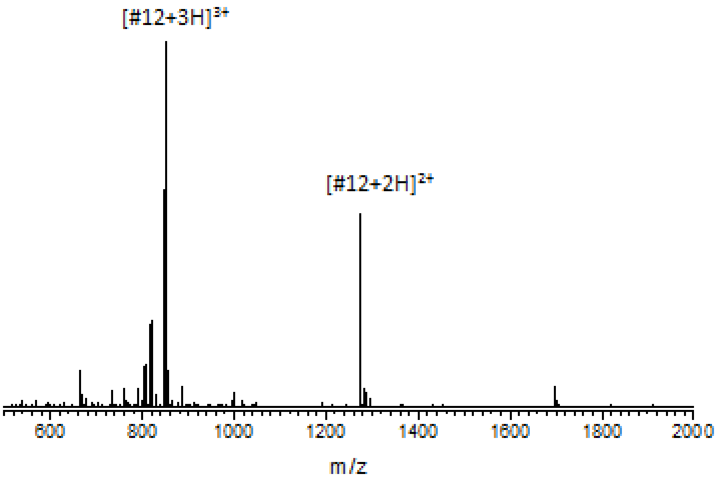

Figure S8: ESI-MS measurements of the product **11.**

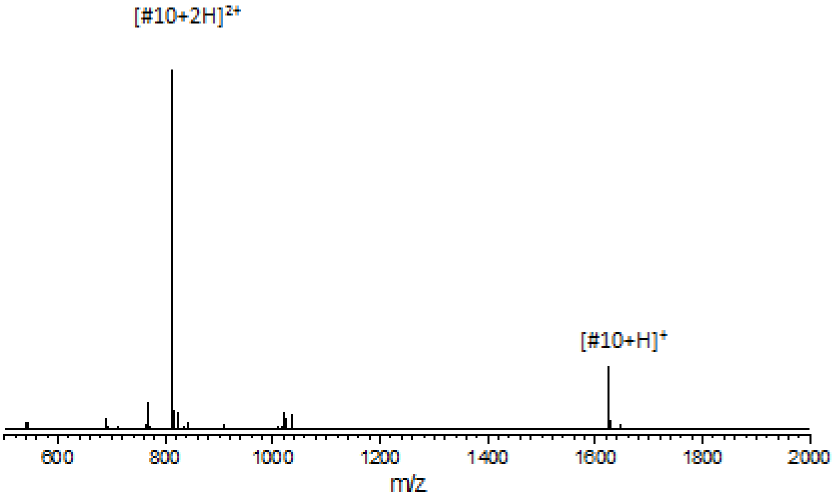

Figure S9: ESI-MS measurements of product **13.**

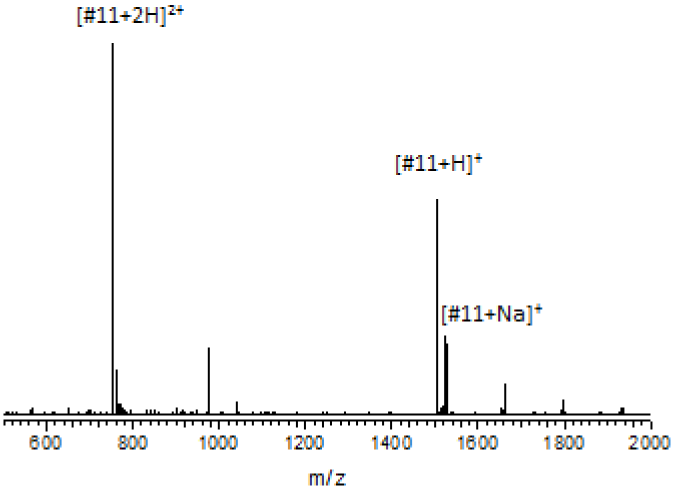

Figure S10: ESI-MS measurements of product **15.**

Switch experiments

For switch experiments *via* NMR, the sample was dissolved in deuterated acetonitrile and stored under exclusion of light. Measurements were performed on a BRUKER 500 spectrometer. ^{1}H signals were recorded with 500 MHz. After the measurement, the sample was radiated for 30 min with UV light and an additional spectrum was accommodated.

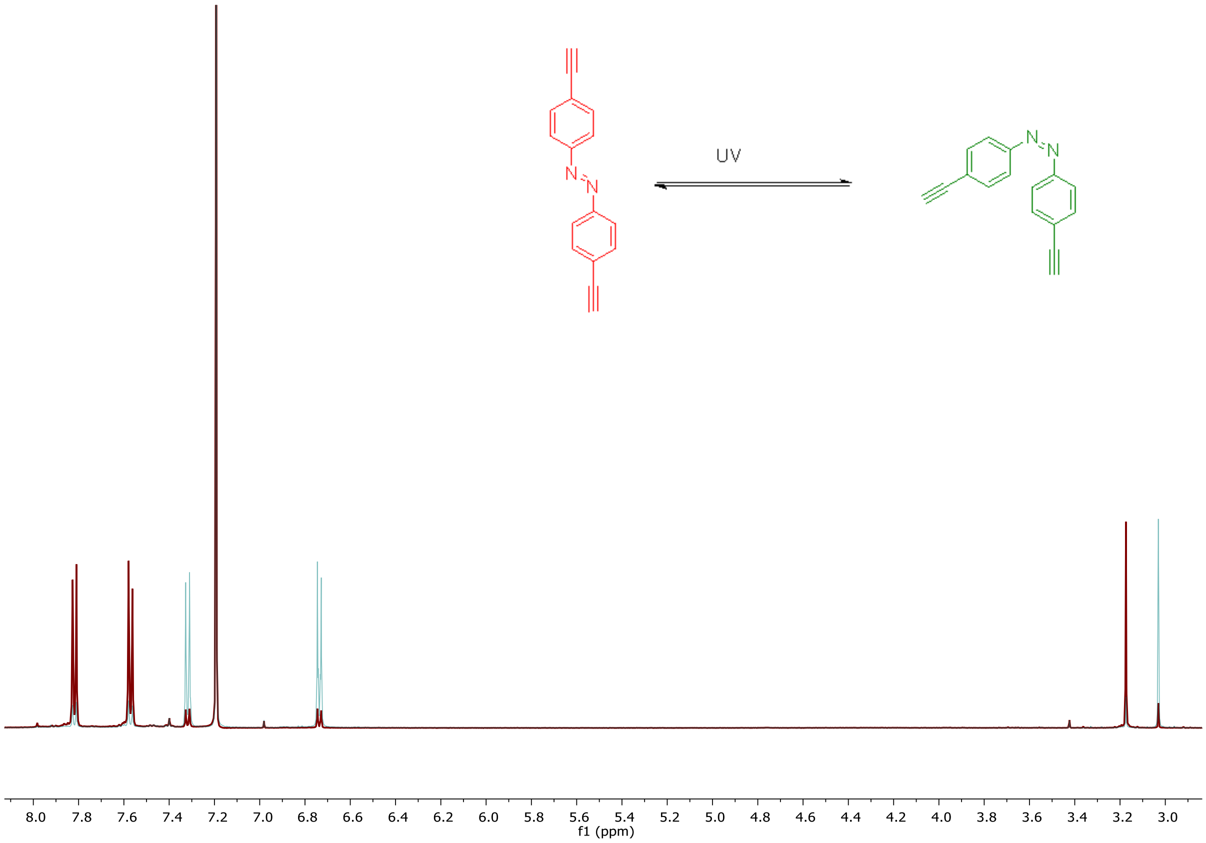

Figure S11: ^{1}H NMR spectrum of compound **21;** red: *trans*-conformation, green: *cis*-conformation.

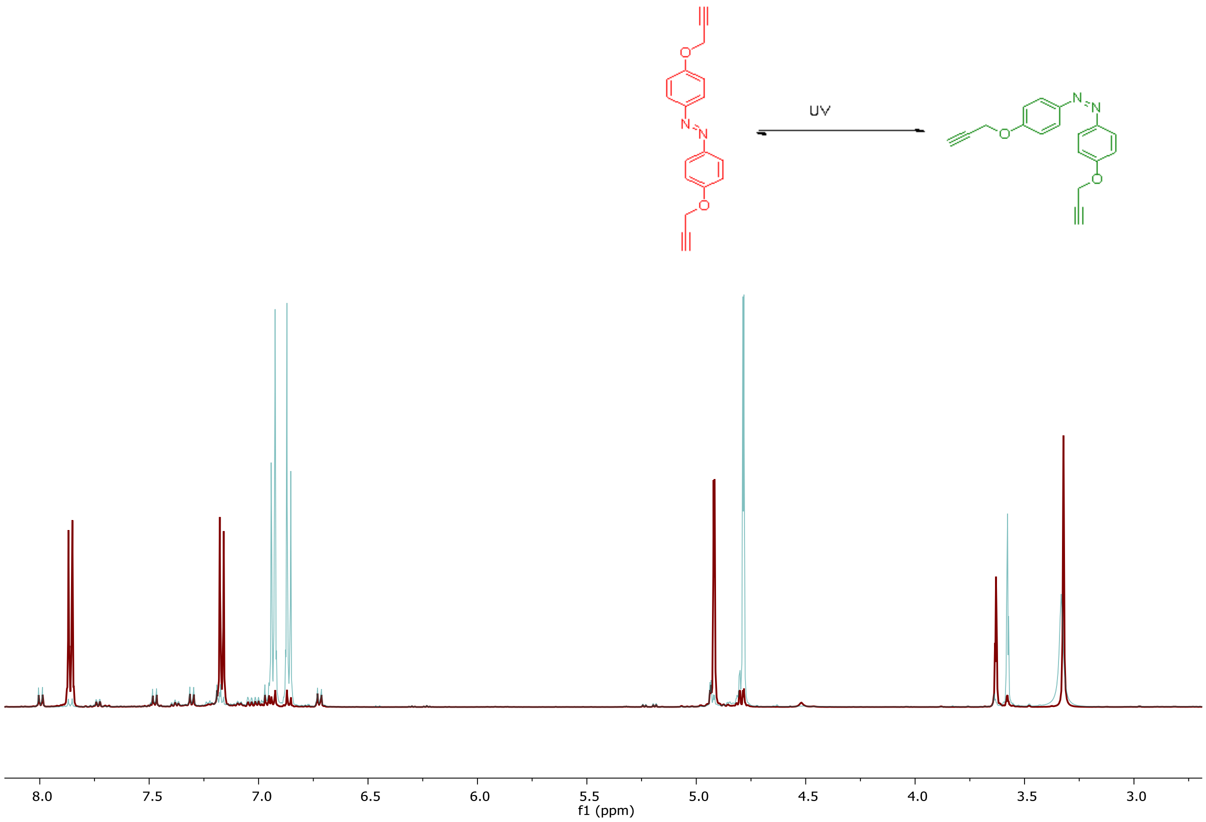

Figure S12: ¹H NMR spectrum of compound **23**; red: *trans*-conformation, green: *cis*-conformation.

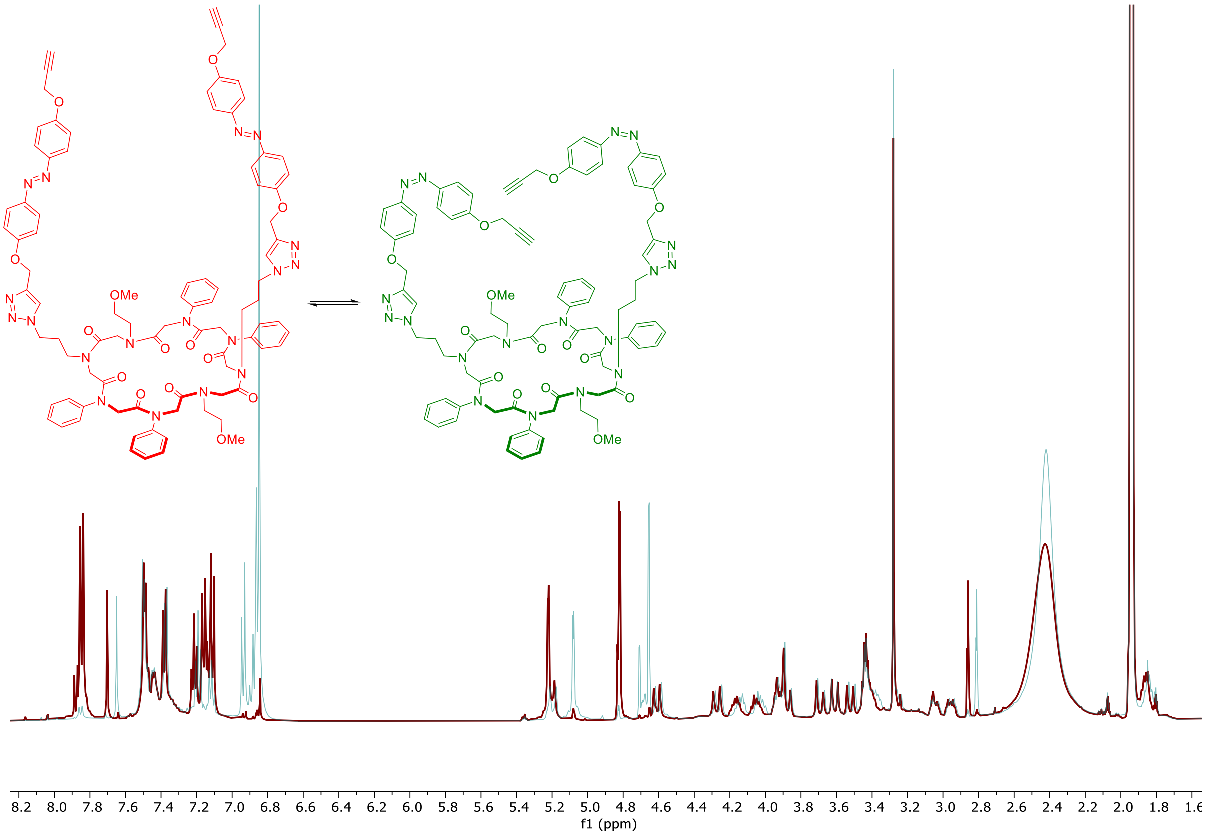

Figure S13: ¹H NMR spectrum of compound **13**; red: trans-conformation, green: cis-conformation**.**

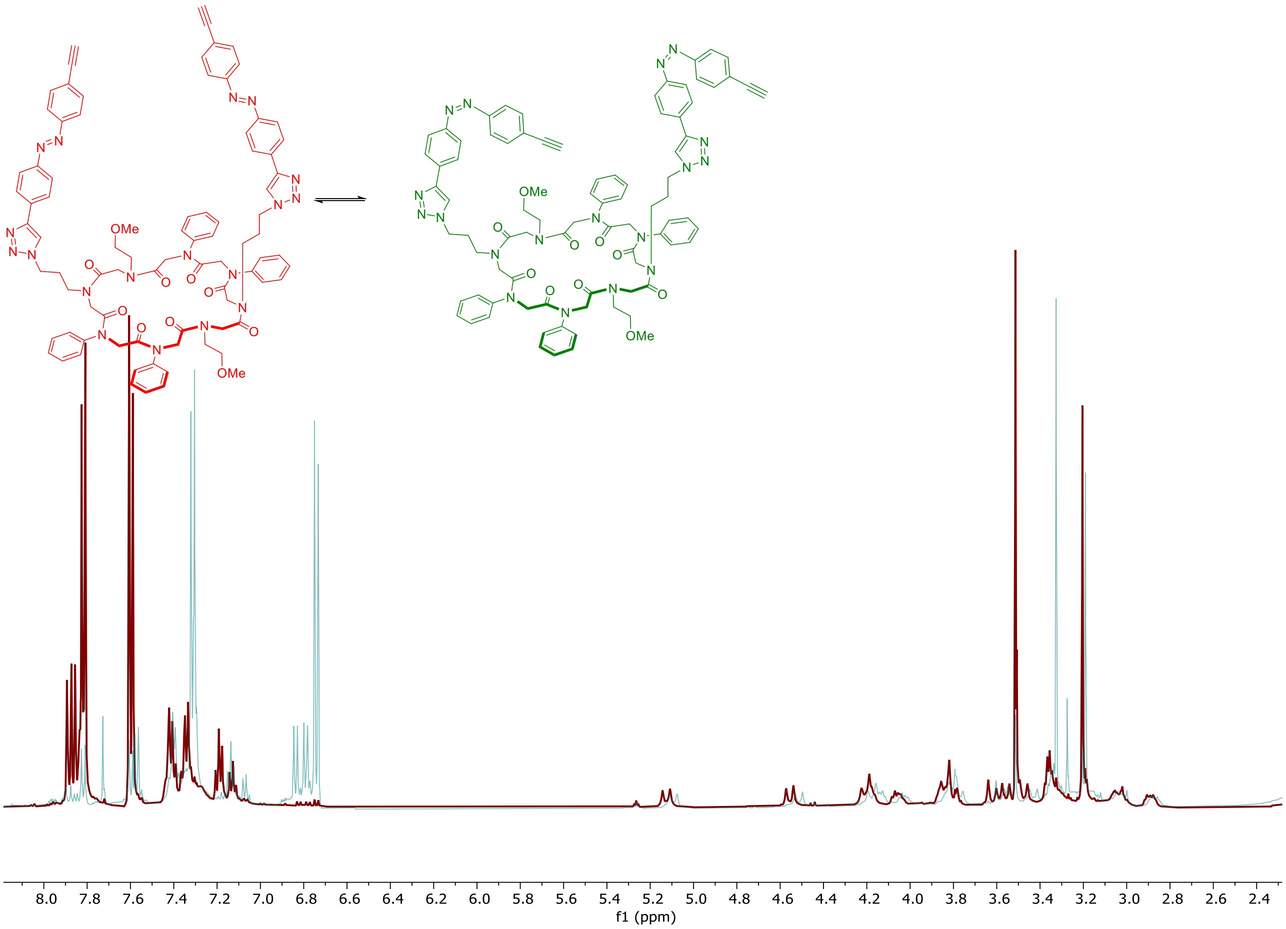

Figure S14: ^{1}H NMR spectrum of compound **15**; red: trans-conformation, green: cis-conformation.

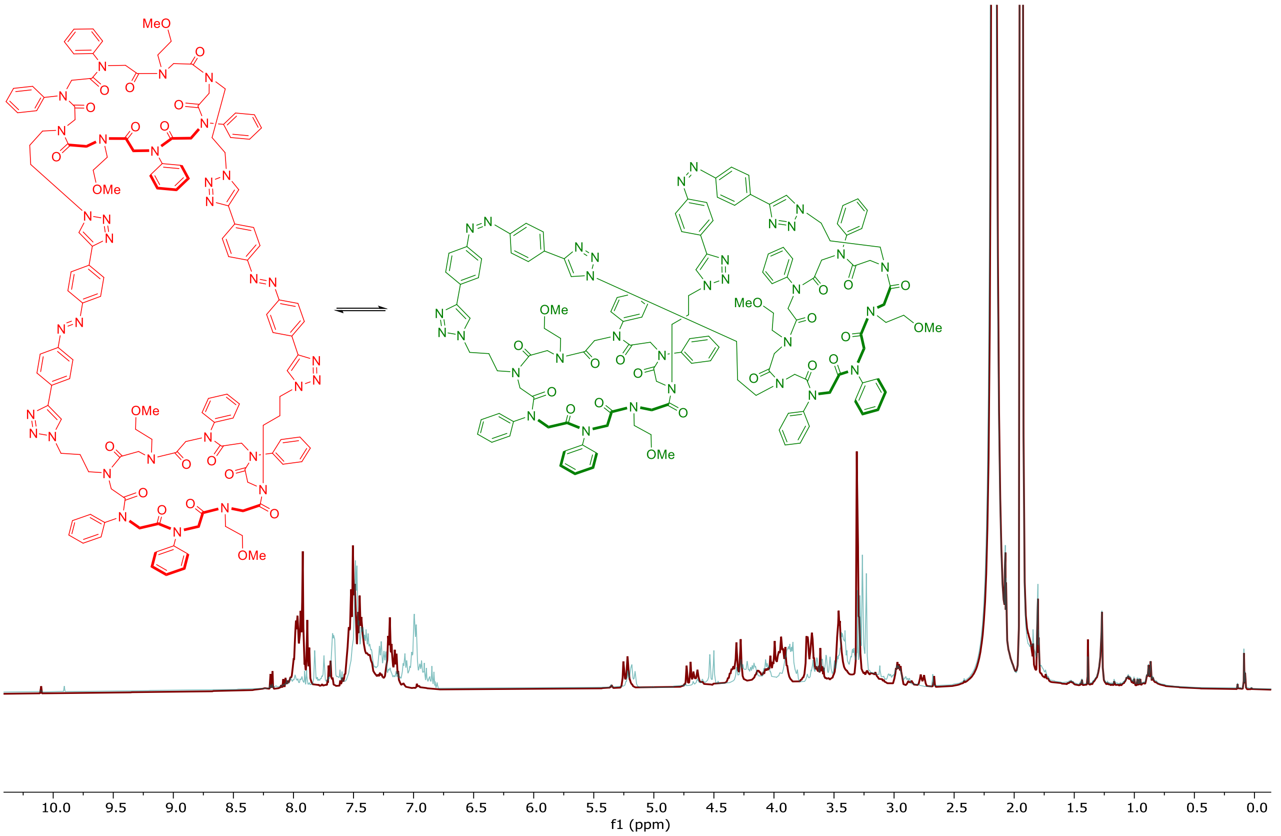

Figure S15: ^{1}H NMR spectrum of compound **16**; red: *trans*-conformation, green: *cis*-conformation.

For switch experiments *via* UV-VIS, a 20 µM solution of each sample was prepared and stored under exclusion of light. Measurements were performed on a LAMBDA 750 spectrometer from PERKINELMER. The spectrum was recorded in the range of 200 nm to 800 nm wavelength. After the measurement, the sample was radiated with UV light (365 nm) for 30 sec and the measurement was repeated. Reversibility was recorded after irradiation with UV light (365 nm) for 1 min first and subsequent irradiation with visible light (460 nm) for 1 min.

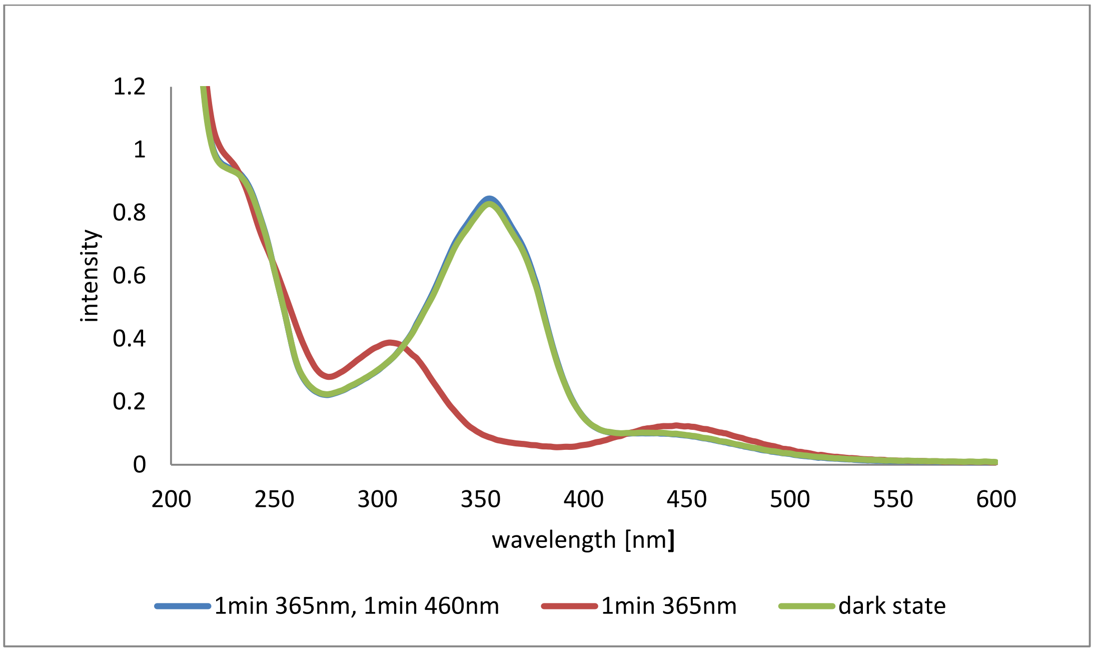

Figure S16: UV-Vis spectra of compound **13**; green, blue: *trans*-conformation, red: *cis*-conformation.

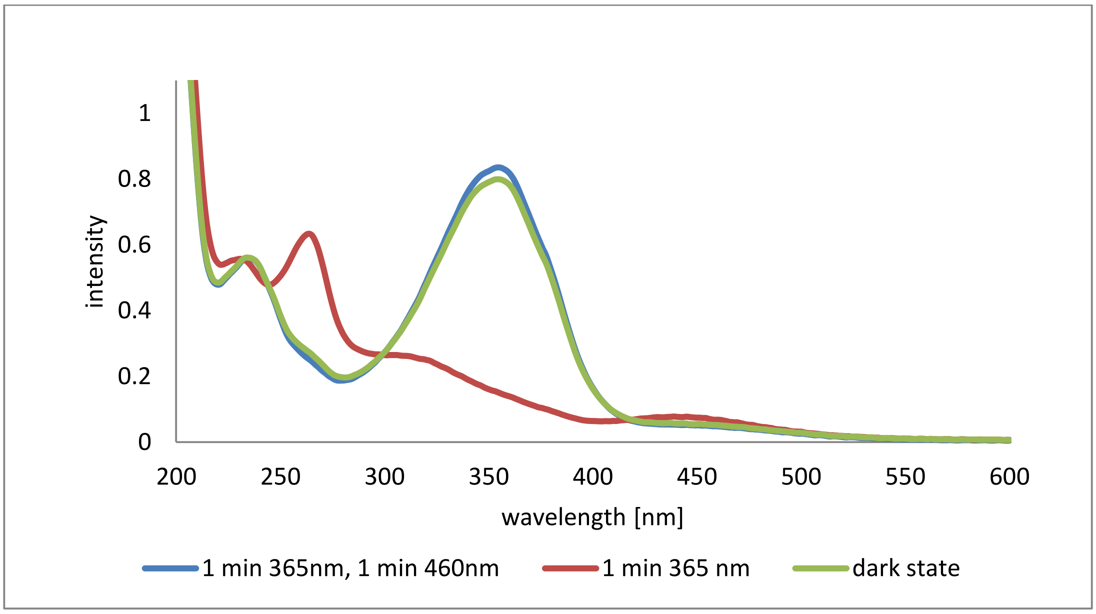

Figure S17: UV-Vis spectra of compound **15**; green, blue: *trans*-conformation, red: *cis*-conformation.

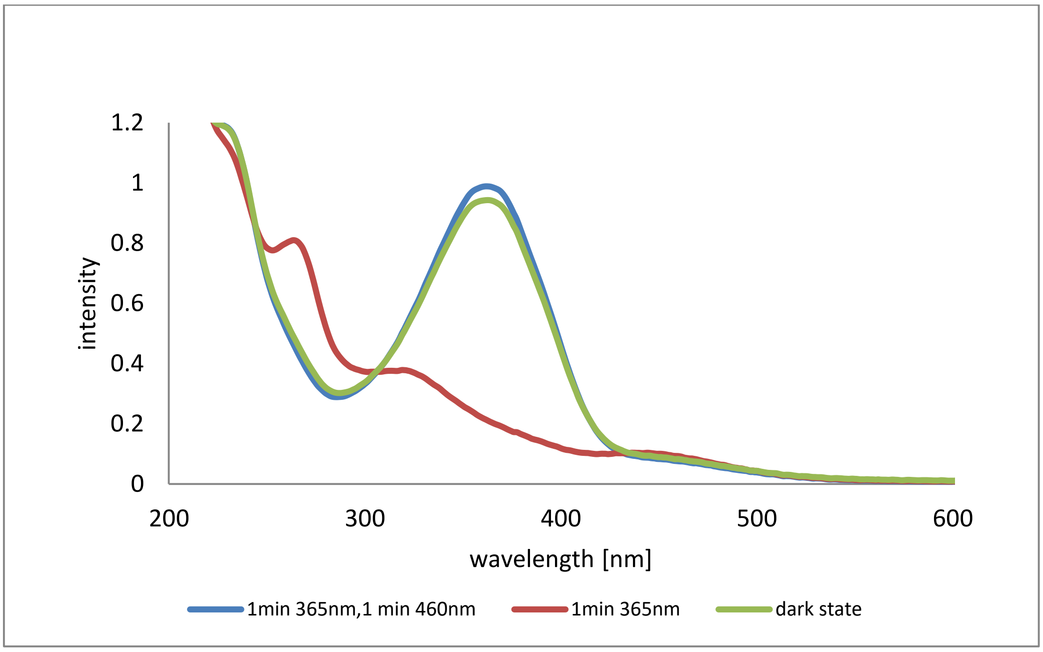

Figure S18: UV-Vis spectra of compound **16**; green, blue: *trans*-conformation, red: *cis*-conformation.

Crystallographic Data

Crystal Structure Determinations

The single-crystal X-ray diffraction study was carried out on a Bruker D8 Venture diffractometer with Photon100 detector at 123(2) K (**7b**) and an Agilent SuperNova-Dual diffractometer with Atlas CCD-detector at 120(2) K (**7b**) using Cu-K -radiation (= 1.54178 Å). Dual Space Methods (SHELXD) (for **7b**) or Direct methods (SHELXS-97) [SHELXD and SHELXS: G. M. Sheldrick, *Acta Crystallogr.* 2008, **A64**, 112-122] (for **7c**) was used for structure solution and refinement was carried out using SHELXL-2013 or SHELXL-2014 (full-matrix least-squares on F^2) [SHELXL: G. M. Sheldrick, *Acta Crystallogr.* 2015, **C71**, 3-8]. Hydrogen atoms were refined using a riding model (H(water) free). Semi-empirical absorption corrections were applied. For **7b** an extinction correction was applied. In **7b** the solvent water and the two 3-azidopropyl groups are disordered (see cif-file for details). In **7c** the absolute structure could not be determined reliably (Parsons x-parameter x = 0.5(2), see cif-file for details) [S. Parson, H.D. Flack, *Acta Crystallogr.* 2004, **A39**, s61].

7b: colorless crystals, $C_{52}H_{62}N_{14}O_{10}$ – 0.5 H_2O, M_r = 1052.16, crystal size 0.36 × 0.12 × 0.06 mm, monoclinic, space group *C2/c* (No. 15), a = 23.0990(10) Å, b = 18.4326(8) Å, c = 25.4929(12) Å, β = 105.159(2)°, V = 10476.5(8) Å^3, Z = 8, ρ = 1.334 Mg/m^{-3}, μ(Cu-K$_\alpha$) = 0.789 mm^{-1}, F(000) = 4456, $2\theta_{max}$ = 145.4°, 50224 reflections, of which 10350 were independent (R_{int} = 0.073), 687 parameters, 51 restraints, R_1 = 0.090 (for 8336 I > 2σ(I)), wR_2 = 0.228 (all data), S = 1.04, largest diff. peak / hole = 1.196 (in disordered 3-azidopropyl) / -0.772 e Å^{-3}.

7c: colorless crystals, $C_{56}H_{64}N_8O_{12}$, M_r = 1041.15, crystal size 0.19 × 0.08 × 0.04 mm, orthorhombic, space group *Pca2₁* (No. 29), a = 36.3213(8) Å, b = 9.1548(3) Å, c = 32.2512(7) Å, V = 10724.0(5) Å^3, Z = 8, ρ = 1.290 Mg/m^{-3}, μ(Cu-K$_\alpha$) = 0.754 mm^{-1}, F(000) = 4416, $2\theta_{max}$ = 152.4°, 24059 reflections, of which 15570 were independent (R_{int} = 0.043), 1369

parameters, 1 restraint, R_1 = 0.079 (for 12810 I > 2σ(I)), wR_2 = 0.214 (all data), S = 1.03, largest diff. peak / hole = 0.851 / -0.306 e Å^{-3}.

CCDC 1446764 (**7b**), and 1446765 (**7c**) contain the supplementary crystallographic data for this paper. These data can be obtained free of charge from The Cambridge Crystallographic Data Centre via www.ccdc.cam.ac.uk/data_request/cif.

a) SHELXD and SHELXS: G. M. Sheldrick, *Acta Crystallogr.* 2008, **A64**, 112-122.

b) SHELXL: G. M. Sheldrick, *Acta Crystallogr.* 2015, **C71**, 3-8.

c) S. Parson, H.D. Flack, *Acta Crystallogr.* 2004, **A39**, s61.

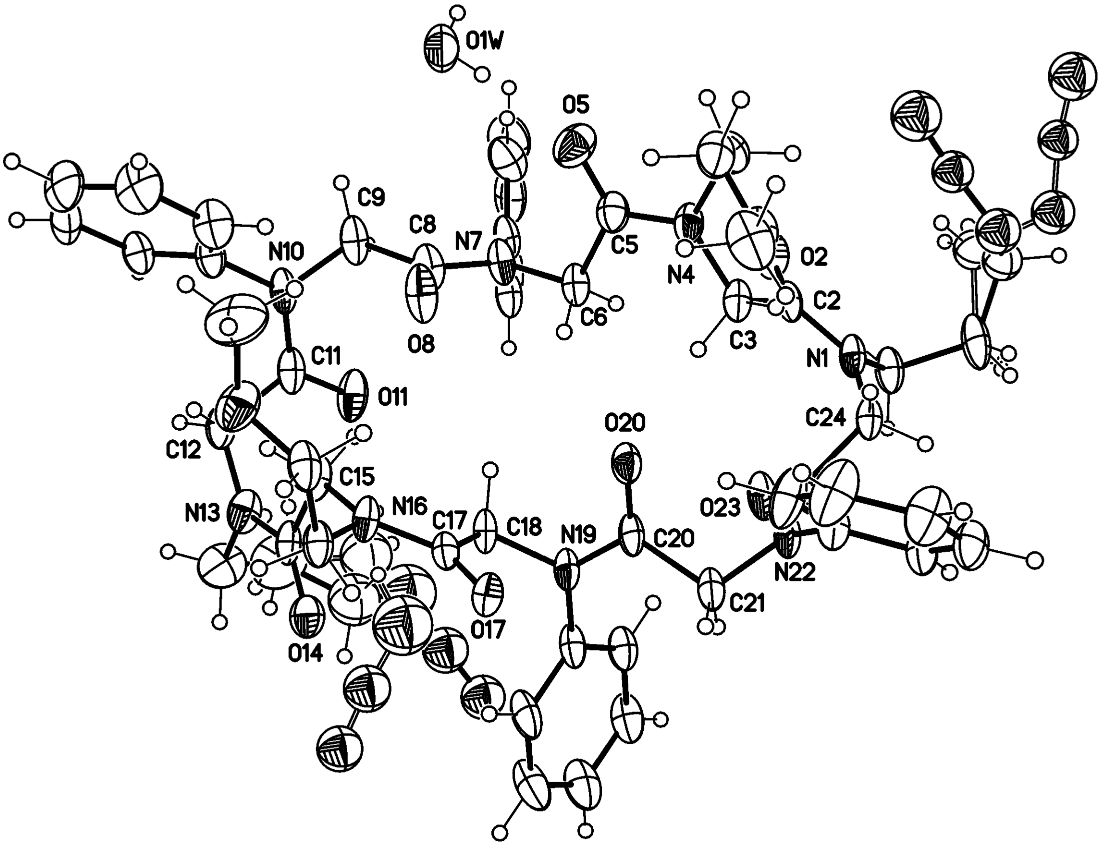

Figure S19. Molecular structure of **7b** (displacement parameters are drawn at 50% probability level).

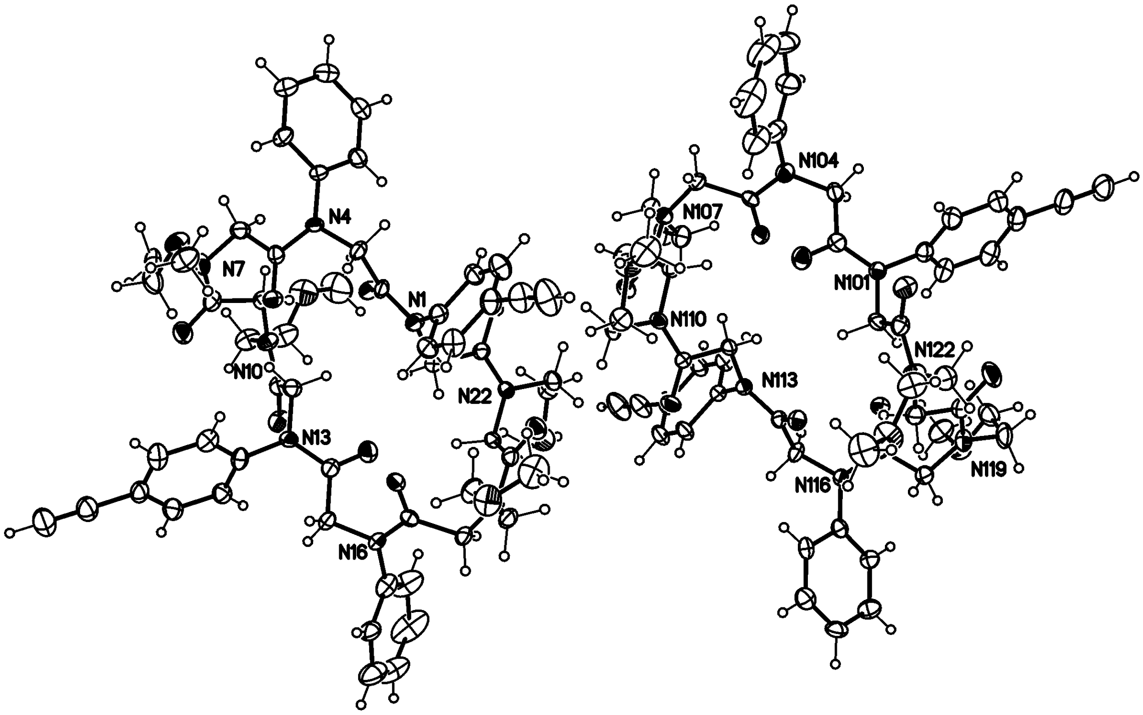

Figure S20. Molecular structure of **7c** (displacement parameters are drawn at 50% probability level).

References

1. B. Burger, J. Bercaw, *ACS Symp.* **1987**, *4*, 79-115.
2. C. Still, M. Kahn, A. Mitra, *J. Org. Chem.* **1978**, *43* (14), 2923-2925.
3. R. N. Zuckermann, j. M. Kerr, S. B. Kent, W. H. Moos, *J. Am. Chem. Soc.* **1992**, *114* (26), 10646-10647.
4. R. B. Merrifield, *J. Am. Chem. Soc.* **1963**, *85* (14), 2149-2154.
5. S. B. Y. Shin, B. Yoo, L. J. Todaro, K. Kirshenbaum, *J. Am. Chem. Soc.* **2007**, *129* (11), 3218-3225.
6. R. Jagasia, J. Holub, M. Bollinger, K. Kirshenbaum, M. G. Finn, *J. Org. Chem.* **2009**, *74* (8), 2964-2974.
7. B. Carboni, A. Benalil, M. Vaultier, *J. Org. Chem.* **1993**, *58* (14), 3736-3741.
8. W. Zhu, D. Ma, *Chem. Commun.* **2004**, 888-889.
9. D. Fürniß, Neue molekulare Transporter auf Polyamin- und Peptoidbasis. *Dissertation, Karlsruher Institut für Technologie (KIT)* **2013**.
10. N. A. Noureldin, J. W. Bellegarde, *Synthesis* **1999**, *1999* (6), 939-942.
11. C. Moreno, A. Arnanz, R.-M. Medina, M.-J. Macazaga, M. Pascual, E. M. García-Frutos, E. Martínez-Gimeno, M.-L. Marcos, *Organometallics* **2015**, *34* (12), 2971-2984.
12. O. Lavastre, I. Illitchev, G. Jegou, P. H. Dixneuf, *J. Am. Chem. Soc.* **2002**, *124* (19), 5278-5279.
13. W. R. Brode, I. L. Seldin, P. E. Spoerri, G. M. Wyman, *J. Am. Chem. Soc.* **1955**, *77* (10), 2 762-2765.
14. C. Kordel, C. S. Popeney, R. Haag, *Chem. Comm.* **2011**, *47* (23), 6584-6586.
15. J. M. Casas-Solvas, M. C. Martos-Maldonado, A. Vargas-Berenguel, *Tetrahedron* **2008**, *64* (48), 10919-10923.
16. S. B. L. Vollrath, C. Hu, S. Bräse, K. Kirshenbaum, *Chem. Commun.* **2013**, *49* (23), 2317-2319.
